# Protein-conformational diseases in childhood: Naturally-occurring hIAPP amyloid-oligomers and early β-cell damage in obesity and diabetes

**DOI:** 10.1371/journal.pone.0237667

**Published:** 2020-08-24

**Authors:** Nelly F. Altamirano-Bustamante, Eulalia Garrido-Magaña, Eugenia Morán, Aurora Calderón, Karina Pasten-Hidalgo, Rosa Angélica Castillo-Rodríguez, Gerardo Rojas, Reyna Lara-Martínez, Edgar Leyva-García, Mateo Larralde-Laborde, Guadalupe Domíguez, Chiharu Murata, Yolanda Margarita-Vazquez, Rafael Payro, Manuel Barbosa, Alejandro Valderrama, Hortencia Montesinos, Alejandra Domínguez-Camacho, Víctor H. García-Olmos, Regina Ferrer, Patricia G. Medina-Bravo, Fernanda Santoscoy, Cristina Revilla-Monsalve, Luis Felipe Jiménez-García, Julio Morán, Jalil Villalobos-Alva, Mario Javier Villalobos, Raúl Calzada-León, Perla Altamirano, Myriam M. Altamirano-Bustamante

**Affiliations:** 1 Instituto Nacional de Pediatría, Mexico City, Mexico; 2 UMAE Hospital de Pediatría, Centro Médico Nacional Siglo XXI, Instituto Mexicano del Seguro Social, Mexico City, Mexico; 3 Unidad de Investigación en Enfermedades Metabólicas, Centro Médico Nacional Siglo XXI, Instituto Mexicano del Seguro Social, Mexico City, Mexico; 4 Cátedras Conacyt, Consejo Nacional de Ciencia y Tecnología, Mexico City, Mexico; 5 Facultad de Ciencias, UNAM, Mexico City, Mexico; 6 Instituto de Fisiología Celular, UNAM, Mexico City, Mexico; 7 Hospital Infantil Federico Gómez, Mexico City, Mexico; University of Akron, UNITED STATES

## Abstract

**Background and aims:**

This is the first time that obesity and diabetes mellitus (DM) as protein conformational diseases (PCD) are reported in children and they are typically diagnosed too late, when β-cell damage is evident. Here we wanted to investigate the level of naturally-ocurring or real (not synthetic) oligomeric aggregates of the human islet amyloid polypeptide (hIAPP) that we called RIAO in sera of pediatric patients with obesity and diabetes. We aimed to reduce the gap between basic biomedical research, clinical practice-health decision making and to explore whether RIAO work as a potential biomarker of early β-cell damage.

**Materials and methods:**

We performed a multicentric collaborative, cross-sectional, analytical, ambispective and blinded study; the RIAO from pretreated samples (PTS) of sera of 146 pediatric patients with obesity or DM and 16 healthy children, were isolated, measured by sound indirect ELISA with novel anti-hIAPP cytotoxic oligomers polyclonal antibody (MEX1). We carried out morphological and functional studied and cluster-clinical data driven analysis.

**Results:**

We demonstrated by western blot, Transmission Electron Microscopy and cell viability experiments that RIAO circulate in the blood and can be measured by ELISA; are elevated in serum of childhood obesity and diabetes; are neurotoxics and works as biomarkers of early β-cell failure. We explored the range of evidence-based medicine clusters that included the RIAO level, which allowed us to classify and stratify the obesity patients with high cardiometabolic risk.

**Conclusions:**

RIAO level increases as the number of complications rises; RIAOs > 3.35 μg/ml is a predictor of changes in the current indicators of β-cell damage. We proposed a novel physio-pathological pathway and shows that PCD affect not only elderly patients but also children. Here we reduced the gap between basic biomedical research, clinical practice and health decision making.

## Introduction

The number of people with DM in the world has almost quadrupled from 1980 to 2014, reaching approximately 422 million people, and it is associated with epidemic obesity [1–3]. The relative annual increase in the incidence of type 1 diabetes (T1DM) in the United States was 1.8% (P<0.001) and of type 2 diabetes (T2DM) was 4.8% (P<0.001) [1]. The expenditures on the treatment of diabetes in 2014 was 612 billion dollars [4].

The diagnosis of T2DM is often made late, when 60% of the pancreas is destroyed and when β-cell damage is evident; targeting the challenge to identify the metabolically unhealthy children with obesity, using a set of 22 cardiometabolic risk factors such central obesity, waist circumference, relative fat distribution, overall body fatness, signs of insulin resistance, measures of blood pressure, triacylglycerols, HDL-cholesterol, insulin, C-peptide, HOMA, ALT, uric acid, microalbuminuria and plasma glucose among others [5,6]. The damage to the islet is due, in part, to the aggregation state (two or more protein unfolded molecules) of naturally-occurring cytotoxic oligomers of islet amyloid polypeptide (hIAPP) [7–17] that we called RIAO (**R**eal h**I**APP **A**myloid **O**ligomers) [18–20]; the aggregation-oligomerization-fibrillization process is active and is well known that there are deposits as amyloid fibrils in more than 90% in patients with T2DM [17,21,22].

In our previous study of *discovery* (isolation, stabilization, initial morphological characterization, and immunoreactivity and biophysical studies on the process of hIAPP aggregation-oligomerization- fibrillation in sera of children with obesity and DM) [20], demonstrated that the RIAO are homo (trimers, hexamers and dodecamers) and hetero-oligomers (main co-aggregation proteins: serum albumin, immunoglobulins and haptoglobin); exist in sera as several sizes (small, medium and large); form fibers and aggregated very fast at 37 °C and delay the process of fiber formation of synthetic hIAPP [20]. As far we know the soluble RIAO level has not been studied in the preclinical and clinical stages of obesity and DM in serum of pediatrics patients. RIAO can be detected by anti-hIAPP and anti-amyloid oligomers (A11) antibodies. Antibodies are very useful tools for disease diagnoses at the level of a protein sequence or conformation. Such methods can track the different stages of the fiber formation process and help construct physio-pathological models of obesity and DM in childhood [9,17,20,21,23,24]. Therefore, there is a great need to improve the gap between basic biomedical research and clinical practice and health decision making in pediatrics.

Our cross-functional project development team accelerates the steps of translating basic scientific discoveries from the laboratory to diabetes clinical applications in childhood. Here we report the step called *RIAO* as a *candidate biomarker of early β-cell failure* that include (i) efficient stabilization (universal pretreatment); (ii) detection (WB); (iii) morphological-functional characterization (TEM and Cell viability assays); (iv) the production and characterization of novel hIAPP anti-oligomer-specific rabbit polyclonal antibody (MEX1); (v) the development of an immunoassay highly reproducible (Indirect Elisa); (vi) human clinical research (pilot study: dissemination and implementation in individual patient care in the three main pediatric hospitals in Mexico city); and (vii) integrating knowledge (evidence-based medicine clusters)]. Furthermore, we constructed different stages of the fiber formation process to generate a novel physio-pathological pathway of obesity and DM as PCD. Our hypothesis is that obesity and DM in children give rise to many PCD and it is a challenge for translational medicine to diagnose, prevent, control, test the transmission mechanisms of and treat PCD.

We consider the shifting of paradigms in obesity and DM, this paradigm changes the way in which PCD are thought of, since it extends the focus from Alzheimer’s disease and other aging diseases towards the pediatric implications of cytotoxic oligomers that are present since childhood, and it opens the epistemic horizon since it shifts our understanding of PCD [18] by showing that they affect not only elderly patients but also children and adolescents.

The financial burden of obesity and diabetes, along with other PCD in the near future, is going to grow exponentially, and the healthcare and socioeconomic systems are going to collapse without a significant improvement in the diagnostic approach, prevention and treatment of obesity and DM as a PCD [1,3,4,25,26].

## Materials and methods

This cross-functional paper of translational medicine uses an amalgam of methods, strategies and ideas that range from organic chemistry, biophysics, biochemistry, to proteomic medicine, landing in the clinical endocrinological field with diseases that are a national and international health problem with a high economic and social cost [1,4,27].

### Study approval

The study protocol (2010-785-052) was approved by the Ethical Committee of Instituto Mexicano del Seguro Social and the Ethical Committee of Instituto Nacional de Pediatría. The study was performed in accordance with the International Harmonization Conference guidelines on Good Clinical Practice. Prior to participation in the study, all parents or legal guardians of the participants provided written informed consent to participate in the study.

The animal protocols, animal care and procedures of the study were approved by the UNAM and IMSS ethical Committee and Animal Care Guideline NOM-062-ZOO 1999. Periodic health evaluations (blood profile, weight, etc.) were made by Veterinary Professionals to ensure that animals were healthy; drinking water and feed were provided *ad libitum*. Methods of euthanasia followed the guidelines given by NOM-062-ZOO 1999 and performed by Veterinary professional; the rabbits were anesthetized with pentobarbital sodic (45 mg/kg) and exsanguinated by cardiac puncture by Veterinary professional. Animals were then euthanized intravenously with pentobarbital sodium (290 mg/kg).

These are acceptable methods by the Vivarium and they were approved by the IMSS ethical committee.

For the cell viability experiments, all animals were obtained from the Vivarium of the Cell Physiology Institute, UNAM. Wistar rat pups of 6 to 8 postnatal days were euthanized by decapitation by personnel with several years of experience, trained and updated with continue education courses in the Academic Vivarium Unity at Instituto de Fisiología Celular, UNAM; following the recommended procedures to avoid unnecessary infliction of pain and using the strictly necessary animals (the total number of rats used per group was 3 newborn Wistar rats 8 post natal day). NGC suspension was obtained from cerebellum of newborn Wistar rats obtained from the Instituto de Fisiología Celular vivarium.

All animal studies were performed in compliance with UNAM Animal Care Guideline NOM-062-ZOO 1999. The study protocol (JMA120-17) was approved by the Ethics Committee of Instituto de Fisiología Celular, UNAM. SAGARPA-SENASICA AUTO-B-C-1216-030.

### Study participants

A multicentric collaborative, cross-sectional, analytical, ambispective, blinded study was carried out.

#### Human subjects and sample collection

Children and adolescents were recruited from three main pediatric hospitals in Mexico: Instituto Nacional de Pediatría (INP) and UMAE-Hospital de Pediatría, Centro Médico Nacional Siglo XXI, Instituto Mexicano del Seguro Social (IMSS) and Hospital Infantil, Federico Gómez. Informed consent was obtained from all participants. The study was conducted with the approval of the Ethics and Research Committees of IMSS and the INP.

The participants were divided into 4 groups (Table 1).

**Table 1:** Clinical characteristics of the participants.

	CTRL (n = 16)	OBS (n = 47)	T2DM (n = 39)	T1DM (n = 60)	P-value	Available case number for each group
Sex [male], n (%)	8 (50%)	24 (51%)	18 (46%)	32 (53%)	0.920	
Age [months]	163 (120, 183)	159 (56, 201)	170 (36, 216)	161 (26, 208)	0.387	
Evolution [months]	---	---	21 (0, 101)	36 (0, 168)	<0.001	(n_T2DM_ = 31, n_T1DM_ = 60)
Height [z-score]	0.34 (-0.36, 1.56)^A^	-0.42 (-2.79, 4.92)^AB^	0.11 (-4.83,1.81)^BC^	-0.56 (-3.09, 1.45)^C^	<0.001	
BMI [z-score]	0.53 (-0.25, 1.00)^C^	1.89 (-0.26, 3.96)^A^	0.56 (-2.24, 3.52)^B^	0.29 (-1.86, 2.40)^C^	<0.001	
zBMI/zHeight [ND]	0.54 (0.39, 0.67)^AB^	0.58 (0.44, 0.80)^A^	0.53 (0.39, 0.70)^B^	0.46 (0.38, 0.56)^C^	<0.001	(n_CTRL_ = 13, n_OBS_ = 43, n_T2DM_ = 33, n_T1DM_ = 58)
SBP [percentile value]	49 (49, 89)^B^	89 (49, 96)^A^	49 (49, 89)^B^	49 (49, 98)^B^	0.006	(n_CTRL_ = 16, n_OBS_ = 42, n_T2DM_ = 30, n_T1DM_ = 53)
DBP [percentile value]	50 (49, 89)	89 (49, 96)	50 (49,94)	89 (49, 98)	0.132	(n_CTRL_ = 16, n_OBS_ = 42, n_T2DM_ = 30, n_T1DM_ = 53)
Glucose [mg/dL]	86 (71, 95)^C^	88 (72, 99)^C^	119 (77, 369)^B^	233 (67, 619)^A^	<0.001	
HbA1c [%]	5.2 (4.5, 6.0)^C^	5.2 (4.2, 6.4)^C^	5.9 (5.0, 13.7)^B^	9.7 (4.8, 14.8)^A^	<0.001	(n_CTRL_3, n_OBS_ = 27 n_T2DM_ = 39, n_T1DM_ = 59)
Insulin [μU/mL]	9.9 (5.9, 17.5)^C^	18.7 (1.7, 76.3)^A^	16.6 (8.4, 24.1)^B^	6.0 (2.9, 9.1)^BC^	<0.001	(n_CTRL_ = 16, n_OBS_ = 43, n_T2DM_ = 9, n_T1DM_ = 2)
C -Peptide [ng/mL]	1.9 (1.4, 3.1)^C^	3.5 (1.4, 6.7)^B^	1.9 (3.4, 0.2, 34)^ABC^	0.3 (0.1, 4.6)^D^	<0.001	(n_CTRL_ = 15, n_OBS_ = 41, n_T2DM_ = 18 n_T1DM_ = 9)
HOMA [score]	1.9 (1.1, 4.0)^B^	4.2 (0.4, 15.7)^A^	5.6 (2.1, 17.8)^A^	2.9 (2.0, 3.8)^AB^	0.022	(n_CTRL_ = 16, n_OBS_ = 44 n_T2DM_ = 10, n_T1DM_ = 2)
QUICKI [score]	0.35 (0.31, 0.37)^A^	0.31 (0.26, 0.46)^B^	0.30 (0.26, 0.34)^B^	0.33 (0.31, 0.34)^AB^	0.041	(n_CTRL_ = 16, n_OBS_ = 45, n_T2DM_ = 9, n_T1DM_ = 2)
Triglycerides [mg/dL]	86 (52, 121)^B^	120 (42, 481)^A^	117 (38, 552)^A^	82 (32, 913)^A^	<0.001	(n_CTRL_ = 16, n_OBS_ = 45, n_T2DM_ = 39, n_T1DM_ = 60)
Total cholesterol [mg/dL]	139 (74, 209)^C^	157 (97, 239)^BC^	163 (100, 312)^AB^	171 (121, 437)^A^	0.008	(n_CTRL_ = 16, n_OBS_ = 45, n_T2DM_ = 39, n_T1DM_ = 60)
HDL [mg/dL]	50 (33, 61)^A^	40 (28, 81)^B^	42 (23, 72)^B^	48 (30, 68)^A^	0.005	(n_CTRL_ = 16, n_OBS_ = 45, n_T2DM_ = 39, n_T1DM_ = 60)
Non-HDL [mg/dL]	95 (40, 149)^C^	112 (60, 188)^AB^	124 (68, 258)^A^	119 (63, 390)^A^	0.001	(n_CTRL_ = 16, n_OBS_ = 45, n_T2DM_ = 39, n_T1DM_ = 60)
LDL [mg/dL]	77 (23, 132)^B^	84 (21, 147)^B^	91 (29, 162)^B^	100 (55, 205)^A^	<0.001	(n_CTR_ = 16, n_OBS_ = 45, n_T2DM_ = 39, n_T1DM_ = 60)
Uric Acid [mg/dL]	4.7 (3.7, 6.8)^AB^	5.7 (2.8, 9.1)^A^	4.9 (2.8, 9.1)^A^	3.9 (2.0, 11.3)^C^	<0.001	(n_CTRL_ = 12, n_OBS_ = 38, n_T2DM_ = 29, n_T1DM_ = 60)
ALT [IU/L]	16 (10, 21)^B^	24 (11, 79)^A^	26 (10, 124)^A^	17 (10, 129)^A^	<0.001	(n_CTRL_ = 11, n_OBS_ = 39, n_T2DM_ = 26, n_T1DM_ = 60)
Urine albumin [mg/L]]	---	3.9 (0.2, 50.5)	6.0 (0.4, 121.9)	3.1 (0.01, 303.2)	0.208	(n_OBS_ = 18, n_T2DM_ = 28, n_T1DM_ = 59)
Physical exercise [yes], n (%)	16 (100%)^A^	21 (47%)^B^	29 (94%)^A^	46 (82%)^A^	<0.001	(n_CTRL_ = 16, n_OBS_ = 45, n_T2DM_ = 31, n_T1DM_ = 56)

CTRL: control group, OBS: obesity group, T2DM: type 2 diabetes mellitus group, T1DM: type 1 diabetes mellitus group, n: sample size. P values were calculated by chi-square teste, if the dependent variable was categorical, and by Welch’s test, if the dependent variable was continuous. The multiple comparison was done by a protected manner. Superscript A, B, C, D indicate the pairs in which the statistical difference were encountered. The pairs do not share the same letter were different al the level of p<0.05

*Diabetes mellitus groups*. Type 1 diabetes mellitus (T1DM): 60 patients. Median age of 161 (26, 217) months; evolution time of the disease 36 (0, 168) months; regular metabolic control (HbA1c 9.7 (4.8, 14.8)%); anti-GAD65 antibody positive.

Type 2 diabetes mellitus (T2DM): 39 adolescents from Mexico city; male adolescents (genital Tanner stage ≥ 2), female adolescents (mammary Tanner stage ≥ 2); aged from 11 years to 16 years 11 months. The evolution time of diabetes mellitus type 2 ranged from 21 (0, 101) months (according to the criteria accepted by the ADA, with fasting glucose ≥ 126 mg/dL or oral glucose tolerance curve with post-CTOG glucose at 2 h ≥ 200 mg/dL, and glycosylated hemoglobin ≥ 6.5%); anti-GAD65 antibody negative; no MODY.

*Obesity group*. Forty-seven adolescents from Mexico City with clinical data on insulin resistance (central obesity, acanthosis, hyperkeratosis); normal glycemia (ADA criteria, normal fasting glycemia, <100 mg/dl and/or post-CTOG glycemia at 2 h <140 mg/dl); glycosylated Hb ≤ 6.5%; no other systemic disease or genetic syndrome; no medication intake; age 159 (56, 201) months years 5 months; BMI ≥ 95th percentile for age and sex [BMI z score 1.89 (-0.26, 3.96) d.e.]; complete lipid profile.

*Control group*. Sixteen healthy adolescents (without apparent acute or chronic disease) were recruited from Mexico City and Zitácuaro, Michoacán; normal fasting glucose <100 mg/dL; HbA1c < 6.5%; no previous history of hyperglycemia. Inclusion criteria: age 9–17 years; BMI between 5th and 84th percentile zs bmi—zs height < = 1 d.e. Exclusion criteria: diabetes mellitus, use of metformin or a mother with gestational diabetes; obesity; any history of malignancy; drug addictions; the use of systemic corticosteroids in pharmacological doses; incomplete laboratory studies.

All the subjects underwent a complete physical examination that included weight measurement, waist circumference, and blood pressure according to the established standards. In addition, a blood sample (after 10 h of fasting) was obtained and stored at -80 degrees.

The following studies were carried out: fasting glucose, fasting basal insulin, C peptide, glycosylated hemoglobin (HbA1c), creatinine, uric acid, free T4 and TSH, AST, ALT, GGT, lipid profile and RIAO (see below).

### Antigen preparation and characterization

hIAPP_1-37_ was purchased from ProteoGenix, France. hIAPP_1-37_ oligomers were prepared essentially as described by Kayed [28,29]. Briefly, 1 mg of hIAPP_1-37_ was solubilized in 400 μL of hexafluoro-2-propanol (HFIP) for 15 min at room temperature. Then, 100 μL of the resulting hIAPP_1-37_ solution was added to 900 μL Milli-Q H_2_O in a siliconized Eppendorf tube. After 20 min of incubation at room temperature, the samples were centrifuged for 15 min at 14,000×g at 4°C, and the supernatant fraction (pH 2.8–3.5) was transferred to a new siliconized tube and subjected to a gentle stream of N_2_ for 10 min to evaporate the HFIP. The samples were then stirred at 500 rpm using a heating block (Thermomixer comfort Eppendorf, USA) for 30 min at 22°C. To prepare highly pure samples, residual trifluoroacetic acid was removed by lyophilization in 0.1 M HCl followed by lyophilization in 50% acetonitrile and storage at -80°C. Then, 10 μL aliquots were taken for observation by TEM. In order to prepare highly pure samples, residual trifluoroacetic acid was removed by lyophilization in 0.1 M HCl followed by lyophilization in 50% acetonitrile and stored at -80°C.

The antigen was characterized by TEM, and its capacity to produce apoptosis was as described previously by Gómez and coworkers [30]. Briefly, for TEM, a 6 μL droplet of the IAPP oligomer reaction was deposited on a 400-mesh copper grid coated with collodion film and allowed to settle for 4 min. The excess solution was wicked away by gently applying a piece of blotting paper to the edge of the grid. Then a 40 μL droplet of 2% uranyl acetate was deposited on the grid and allowed to settle for 60 s. The excess solution was removed as mentioned above. The grid was air-dried for 24 h to be later observed using a JEM-1010 JEOL (Japan) microscope operated at an acceleration voltage of 80 kV. The electronic micrographs were acquired using an MTI model CCD-300-RC camera (Japan). All of the experiments were performed at least three times, and representative samples are shown in results.

The ability of synthetic hIAPP oligomers to produce neurotoxicity in cell culture was examined in cerebellar granule cells (CGCs) as reported by Gomez [30]. Briefly, cells were seeded on a 48-well plate at 1.5 x 10^5^ cells/mL. The next day, the medium was replaced with fresh medium containing freshly dissolved hIAPP oligomers (80 μmol/L). Twenty-four hours later, MTT (0.1 mg/mL) was added to the CGNs and incubated for 15 min at 37°C. After the removal of medium containing MTT, 100% DMSO was added to the dishes and incubated for 15 min at room temperature in darkness. Formazan blue formation was measured at 560 nm in an ELISA plate reader (microplates ELx800, BioTek). Values were corrected to the background signal.

### Antibody production and characterization of conformational specificity

The synthetic hIAPP oligomers (antigen) were used to immunize one New Zealand White female rabbit (from UNAM’s bioterium) in compliance with IMSS Animal Care Guideline NOM-062-ZOO 1999. The rabbit was immunized with 0.75 mg of antigen in complete Freund’s adjuvant. The injected sample was monitored by TEM, followed by boosting three times at 1-wk intervals with 0.75 mg of antigen in Freund’s adjuvant. The polyclonal antibody was partially purified by ammonium sulfate precipitation, dialyzed, concentrated with glycerol and stored at -20°C. The specificity of the novel polyclonal antibody was determined by biochemical analysis (Western blot, dot blot, and ELISA) using well-characterized hIAPP monomer, hIAPP oligomer and hIAPP fibril samples and was compared with purified anti-amyloid oligomer antibody ab126892 (A11) (Abcam, cat: AB 126892)^4^ and anti-amylin antibody (Santa Cruz Biotechnology, cat SC-57026).

All the animals were obtained from the UNAM´s Vivarium; New Zealand White female rabbits were immunized with the antigen (0.75 mg) in complete Freund’s adjuvant. followed by boosting three times at 1-wk intervals with 0.75 mg of antigen in Freund’s adjuvant.

Animals were injected subcutaneously in small increments of 0.1 ml per site in a checkerboard fashion on the scapular region; following the recommended procedures to avoid unnecessary infliction of pain and using the strictly necessary animals (2 rabbits). All animal studies were carried out in strict accordance with the guidelines of UNAM Animal Care and IMSS Animal Care Guideline NOM-062-ZOO 1999. All the animals were from the same line and reared in similar conditions at the Breeding Station of UNAM Vivarium and were sacrificed according to the standards of the Animal Management of UNAM and IMSS guidelines: the rabbits were anesthetized with pentobarbital sodic (45 mg/kg) and exsanguinated by cardiac puncture by Veterinary professional. Animals were then euthanized intravenously with pentobarbital sodium (290 mg/kg). The study protocol was approved by the Ethical Committee of IMSS.

### Enzyme-Linked Immunosorbent Assay (ELISA)

Pretreated samples (PTS) were prepared as previously described [20], briefly 1000 μL of each serum sample is mixed with 9000 μL of ice-cold methanol/acetic acid solution (33% methanol,4% acetic acid) for 1.5 h at 4 °C in order to precipitate the oligomers; then pelleted and resuspended, as described by Bram and coworkers [31]. PTS were applied in duplicate to 96-well plates and analyzed by ELISA using the novel anti-hIAPP-cytotoxic oligomer antibody (MEX1) prepared in house following the modified Kayed method [28]. In brief, the plates were incubated for 24 h at 4°C, washed three times with PBS-T (PBS containing 0.01% Tween 20) and then blocked at room temperature for 2 h with 3% BSA/TBS-T (Tris-buffered saline containing 0.01% Tween 20). The BSA used was IgG free (Sigma). The plates were then washed three times with PBS-T, and 100 μl of anti-IAPP oligomer (1:800 dilution in 3% BSA/TBS-T) was added and incubated overnight at 4°C. The plates were washed three times with PBS-T. Then 100 μl horseradish peroxidase-conjugated anti-rabbit IgG (Sta. Cruz diluted 1:20,000 in 3% BSA/TBS-T) was added, followed by incubation for 2 h at RT. The plates were washed three times with PBS-T and developed using 3,3′,5,5′-tetramethylbenzidine (TMB, Sigma, Mexico.). The reaction was stopped with 100 μL of 2 M H_2_SO_4_, and the plates were read at 450 nm. The measurements were taken blinded to the diagnoses.

### Validation of the standard curve

Synthetic hIAPP_1-37_ oligomer standards prepared as described by Kayed were reconstituted in 500 μl of 0.01% NH_4_OH, sonicated in iced water for 10 min and then diluted 1:2 in 50 mM sodium bicarbonate pH 9.0 to achieve the desired concentration of antigen. The antigen was characterized by TEM and confirm its stability in 0.1% NH_4_OH (final pH 9.5–10.5) and stored a 4°C for months based on TEM and immunoassay experiments as described by our group and Kayed for many amyloid oligomers [20,29,32]. We made serial dilutions of the hIAPP_1-37_ oligomer standard from 0.01 μg/μl. The standard curve of each ELISA plate was drawn. The absorbance results of the plate were exported to an Excel spreadsheet, where each absorbance was given its corresponding sample code and the standard-curve absorbance was matched with its corresponding concentration. These results were then exported to GraphPad Prism 7 or Origin Lab 8.0, where the standard-curve concentrations were transformed by log_2_. This transformed curve was now used to interpolate the absorbance results of the samples, and the concentrations were obtained and then analyzed. The curve was adjusted to a polynomial of order 3 (y = ax^3^+bx^2^+cx+d).

We determined the accuracy and reproducibility of the standard curve (running in triplicate) with 12 independent assays during the experimentation (on different plates on different days) under one single transformation measurement (approximately 1 year). The examples shown in results are some of the curves made, and we found consistency between plates, as shown by the inter- and intra-assay variability.

### Western blot

Total protein concentration of oligomers from PTS from the sera of either controls or patients included in the study was determined by BCA method, and the concentration of the samples was normalized. WB assays were done as previously described [9] briefly the PTS were loaded (30 μg) and run in 15% bis-tris SDS-PAGE gels. The membranes were probed overnight at 4°C with the novel anti-hIAPP oligomer antibody (MEX1) (1:800) or the purified anti-amyloid oligomer antibody ab126892-A11 (Abcam, cat AB-126892 1:1,000) or anti-amylin antibody (Santa Cruz Biotechnology, cat. SC-57026, 1:200) diluted in phosphate-buffered saline with Tween-20 (PBS-T). To control the charge, we performed immunoblotting with anti-transferrin (Santa Cruz Biotechnology, cat. sc-30159; 1:500). The membrane activity was detected by substrate chemiluminescence (Immobilion Chemiluminescence HRP Substrate 1:1) and revealed by a Li-COR C-DiGit system. The intensity of the protein bands was quantified by Image Studio Lite via scanning densitometry. The data were managed with MS Excel, while the statistics and graphs were obtained with the SAS JMP 9 statistical software package.

### Cell viability [30,33]

Cell culture: Neuronal granule cells (NGCs) were cultivated in BME (Sigma) supplemented with 10% fetal bovine serum (FBS Biowest) and antibiotics (100 U/ml penicillin and 10^−6^ μg/ml streptomycin; GIBCO). Primary NGC cultures were obtained following Moran and Patel’s protocol [33]. Briefly, a 7–8 post natal day, newborn rat NGC suspension (D99/2019) was obtained from cerebellum from the Instituto de Fisiología Celular vivarium, UNAM (protocol number JMA120-17). The neurons were placed in a 24-well plate previously treated with poli-L-lysine for 24 h. Then, arabinose cytosine (10 μM) was added to prevent nonneuronal cell growth. Cell cultures were kept at 37°C in a humid, water vapor-filled atmosphere with 5% CO2 and 95% air for 8 d until use.

Plates were divided into 2 groups to measure cell viability with two different methods. After 7 d of cultivation, some wells were treated with RIAO from PTS at two concentrations (7.5 μl/300 μl or 15 μl/300 μl). Some other wells remained in their original medium, while in others, their medium of KCl 25 mM was replaced with KCl 5 mM to induce neuronal apoptosis.

After 7 DIV, a group of cells was incubated with 3-(4,5-dimethylthiazol-2-yl)-2,5-diphelyltetrazolium bromide (MTT) for 10 min. Then the medium was removed, and DMSO was added.

The other cell group, kept under the same conditions for 25 h, was treated with calcein-AM (0.4 M) and propidium iodide (IP; 2 μM) for 10 min. Pictures were taken with an epifluorescence microscope, and both calcein-positive (metabolically active) and IP-positive cells were quantified in 2 fields/well. The sum of cells positive for either staining was taken as the total number of cells.

All the animals were obtained from the Vivarium of the Cell Physiology Institute, University of Mexico. Wistar rat pups of 6 to 8 postnatal days were euthanized by decapitation by personnel with several years of experience, trained and updated with continue education courses in the Academic Vivarium Unity at Instituto de Fisiología Celular, UNAM; following the recommended procedures to avoid unnecessary infliction of pain and using the strictly necessary animals (the total number of rats used per group was 3 newborn Wistar rats 8 post natal day).

All animal studies were performed in compliance with UNAM Animal Care Guideline NOM-062-ZOO 1999. The study protocol was approved by the Ethical Committee of Instituto de Fisiología Celular, Universidad Nacional Autónoma de México (protocol number JMA120-17); UNAM. SAGARPA-SENASICA AUTO-B-C-1216-030.

### Statistics

The distribution of RIAO values in the four established groups (healthy children, children with obesity, children with T2DM and children with T1DM) was plotted in histograms to visually compare the distributions.

The clinical-biochemical characteristics of the subjects in the four groups are summarized in results. The categorical variables are listed as the number of cases and their percentage within each group. The quantitative variables are described by the median with the minimum and maximum values due to the lack of compatibility between the majority of the studied variables. The difference between the groups was determined by the chi-square test for categorical variables and by the Welch test for others. To identify cases with a high risk of damaged β-cells, a hierarchical cluster analysis was performed by the application of the Ward method in the group of children with obesity using the following variables: height/waist ratio, TAS, TAD, C-peptide, HOMA, QUICKI, TG, HDL, non-HDL, AU, ALT and regular performance of the recommended exercise. These twelve variables were selected by the presence of an association with the current level of cytotoxic oligomer and data availability. Different numbers of clusters (from two to five) and the gathering of the cases into three clusters were chosen for better interpretability of the presentation. The clinical-biochemical profile of the three clusters was interpreted by examining the behavior of staged data. Among the clusters obtained, the RIAO level was compared by means of the Welch test [34].

Once the adolescents with obesity and high risk of β-cell damage were determined, where the dependent variable was “risk for damage of beta cells” and the independent variable was the RIAO levels, an analysis of association by logistic regression was performed. Additionally, an ROC curve was built using the logistic regression model, and the diagnostic utility was evaluated using the quantification of the area under the ROC curve (AUC). The cut point that optimized the relationship of sensitivity and specificity was identified. Using the identified cut point, the following parameters of diagnostic utility were estimated: sensitivity, specificity, positive likelihood ratio and negative likelihood ratio.

In order to estimate the effect of an increase in the RIAO value on the increase in the risk of damage to beta cells, the odds ratio was calculated for each one-unit change in RIAO and for the identified risk group, based on the local regression models. The RIAO levels were taken either as an independent quantitative value (RIAO level) or as a qualitative variable (group of children with RIAO above the cut point).

In both categorical variables and continuous numeric variables, multiple comparisons were performed in a protected manner. This means that when three or more groups were compared, with the variables that presented a statistically significant difference, a comparison between all the pairs of groups was carried out to determine where the differences were found.

In all the statistical tests, statistical significance was recognized at a level of p<0.05. All the estimated parameters are presented with a confidence interval of 95%. The statistical analysis was carried out with JMP11 software (SAS Institute).

## Results

### Study participants

A total of 146 pediatrics patients and 16 healthy children as controls were included: Group A: T1DM, 60 patients; group B: T2DM, 39 patients; group C: obesity, 47 patients; and group D: 16 healthy children. Their clinical findings are summarized in Table 1.

### Generation of novel anti-hIAPP cytotoxic oligomer antibody (MEX1) as a tool to quantify naturally-occurring or Real hIAPP Amyloid Oligomers (RIAO) in pretreated samples of sera of children with obesity or diabetes

RIAO were precipitated from pretreated samples of sera (PTS) of children with obesity, T1DM, and T2DM [16,20]. We found that RIAO are very stable in 0.1% NH_4_OH (final pH 9.5–10.5) and stored a 4°C for months based on Transmission Electron Microscopy (TEM) and immunoassay experiments as described by our group and several groups for many amyloid oligomers [20,29,32].

**Fig 1:**
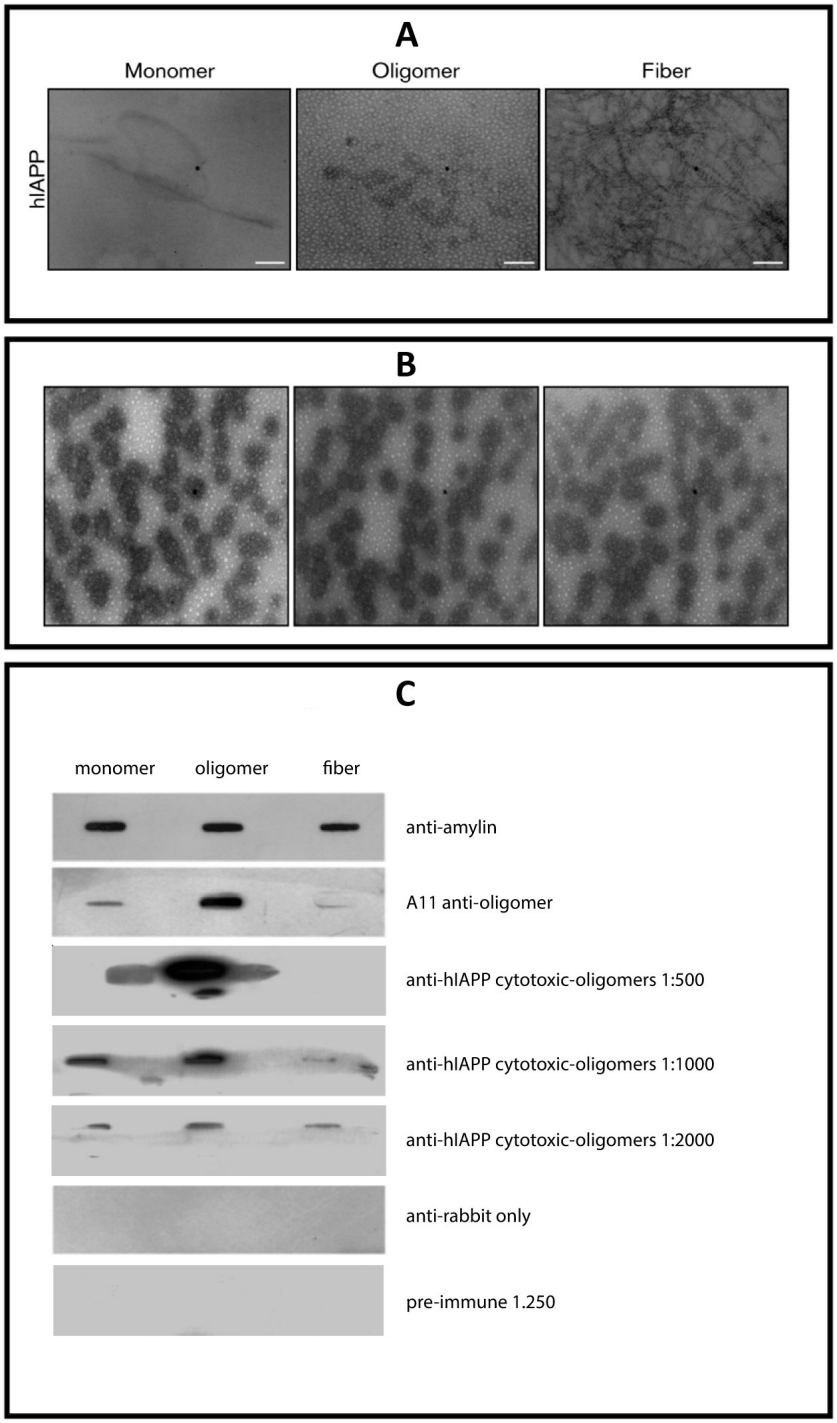
Production and characterization of novel polyclonal anti-hIAPP cytotoxic oligomers antibody (MEX1). A). Representative negative-stain TEM images of monomers, spherical and cytotoxic oligomers and fibrils of synthetic hIAPP used in the production and characterization experiments of the novel polyclonal antibody. Scale bar, 200 nm. B). Antigen characterization. Representative electron microscopy images of the antigen and adjuvant used in the production of anti-hIAPP cytotoxic oligomer polyclonal antibodies. C). Slot blot analysis of monomers, oligomers and fibrils of synthetic hIAPP. Anti-amylin antibody binds to the amino terminus of the three species (monomer, oligomers and fibrils). hIAPP oligomers only stain with anti-oligomer (A11) antibody and the novel anti-hIAPP cytotoxic oligomer polyclonal antibody (MEX1). The synthetic hIAPP oligomers reacted with MEX1 which indicates that is specific to the oligomeric conformation and has no reactivity toward monomers or fibrils.

We used a novel anti-hIAPP cytotoxic-oligomers polyclonal antibody (MEX1) produced by our research group. The specificity of the novel polyclonal antibody was determined by biochemical analysis (Western blot, dot blot, and ELISA) using well-characterized hIAPP monomer, hIAPP oligomer and hIAPP fibril samples and was compared with purified anti-amyloid oligomer antibody ab126892 (A11) (Abcam)^4^ and anti-amylin antibody (Santa Cruz Biotechnology) (Fig 1A–1C).

**Fig 2:**
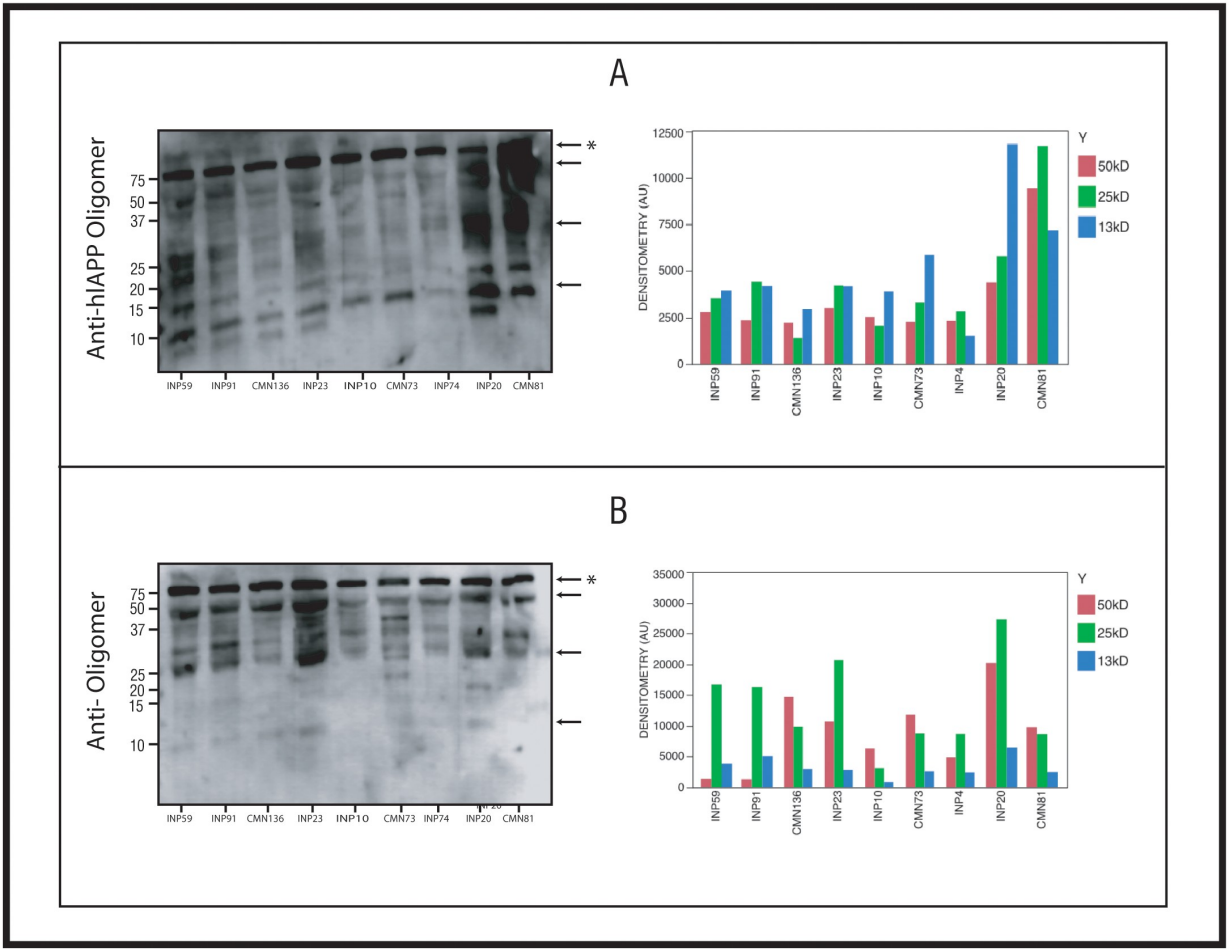
Riao characterization. Western blot analysis of PTS of pediatric patients (30 μg of total protein/lane. A) PTS reacting to the anti-hIAPP cytotoxic oligomer polyclonal antibody. B) PTS reacting to the anti-oligomers (A11). Codes of samples: INP59:T1DM, INP91:T1DM, CMN136: Healthy child, INP23:T1DM, INP10: T1DM, CMN73:T2DM, INP4:T1DM, INP20:T1DM, CMN81:obesity. Detection of transferrin was used as a loading control (*). The numbers on the left correspond to the molecular weight of the bands (kDa). The 50 kDa and 25 kDa markers were the most abundant. Densitometric analysis of the samples for the kDa bands of 50, 25, and 13 kDa. The amount of RIAO found in each of these bands for these three molecular weights is shown.

We demonstrated the presence of RIAO by Western blotting (WB) using the anti-hIAPP cytotoxic oligomer antibody (MEX1) prepared in house and with the anti-amyloid oligomer antibody (A11); both MEX1 and A11 reacted with prefibrillar and cytotoxic oligomers (Fig 1C) and recognize the typical generic epitope to the oligomer conformation [20,28,35] (Fig 2A and 2B). RIAO exhibited several aggregation-oligomerization states; the anti-hIAPP cytotoxic oligomer immunoreactivity showed trimers (13 kDa), hexamers (25 kDa) and dodecamers (50 kDa) (Fig 2A). In the case of hIAPP oligomers range from 3–18 mer; the trimers are well stained by MEX1 since it was obtained in response to immunization with synthetic hIAPP cytotoxic oligomers. Hexamers (25 kDa) and dodecamers (50 kDa) were found with anti-amyloid oligomer antibody (A11) (Fig 2B). The densitometric distribution differences in the reactivity of the PTS with MEX1 and A11 antibodies, revealed that the antibody variants can exhibit differences in RIAO binding and staining by using patient´s samples [36,37]. Densitometric quantification of each of the oligomerization states showed that the amounts significantly varied in RIAO between obesity, T1DM or T2DM and healthy children (Fig 2A and 2B).

**Fig 3:**
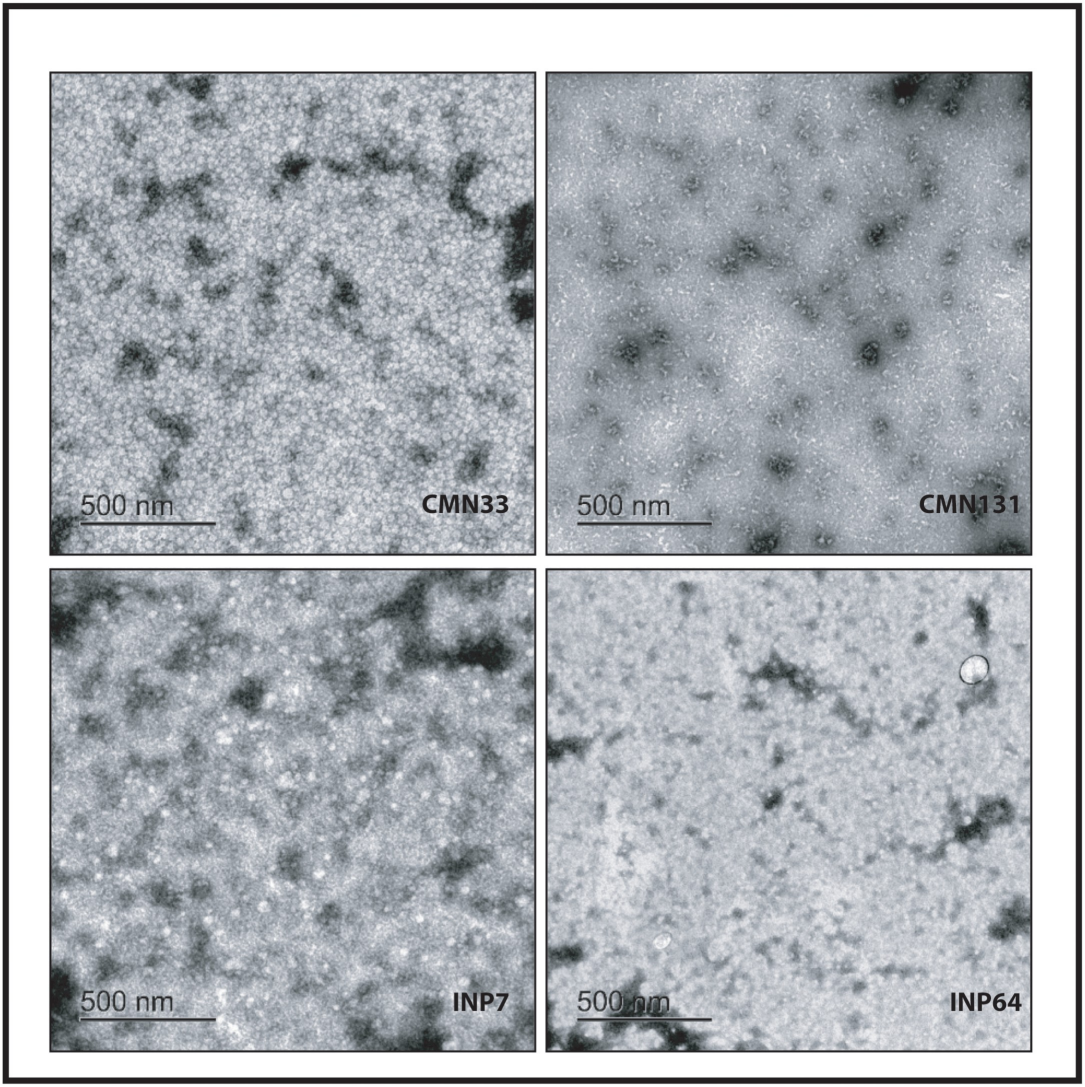
Representative electron microscopy images of RIAO from PTS of all the study groups. Codes of samples: CMN33:obesity; INP7:T1DM; INP64:T2DM; CMN131:healthy child.

The morphology of the RIAO from PTS of pediatric patients from the study population was analyzed by TEM. As seen in the PTS from three patients and one control in Fig 3, we observed the presence of large oligomers and some small fibers (CMN33-obesity); in the sample CMN131-healthy child, we noticed the absence of oligomers, whereas in the sample INP7-T1DM1, there was the presence of large, medium and small oligomers. It looked like the oligomers unite and open to form wide and long fibers. However, there were also small fibers. In sample INP64-T2DM, there were small oligomers with mixed small fibers, and a net formed out of the oligomers’ fibers.

### Neurotoxicity of RIAO from PTS of children and adolescents with obesity or diabetes

**Fig 4:**
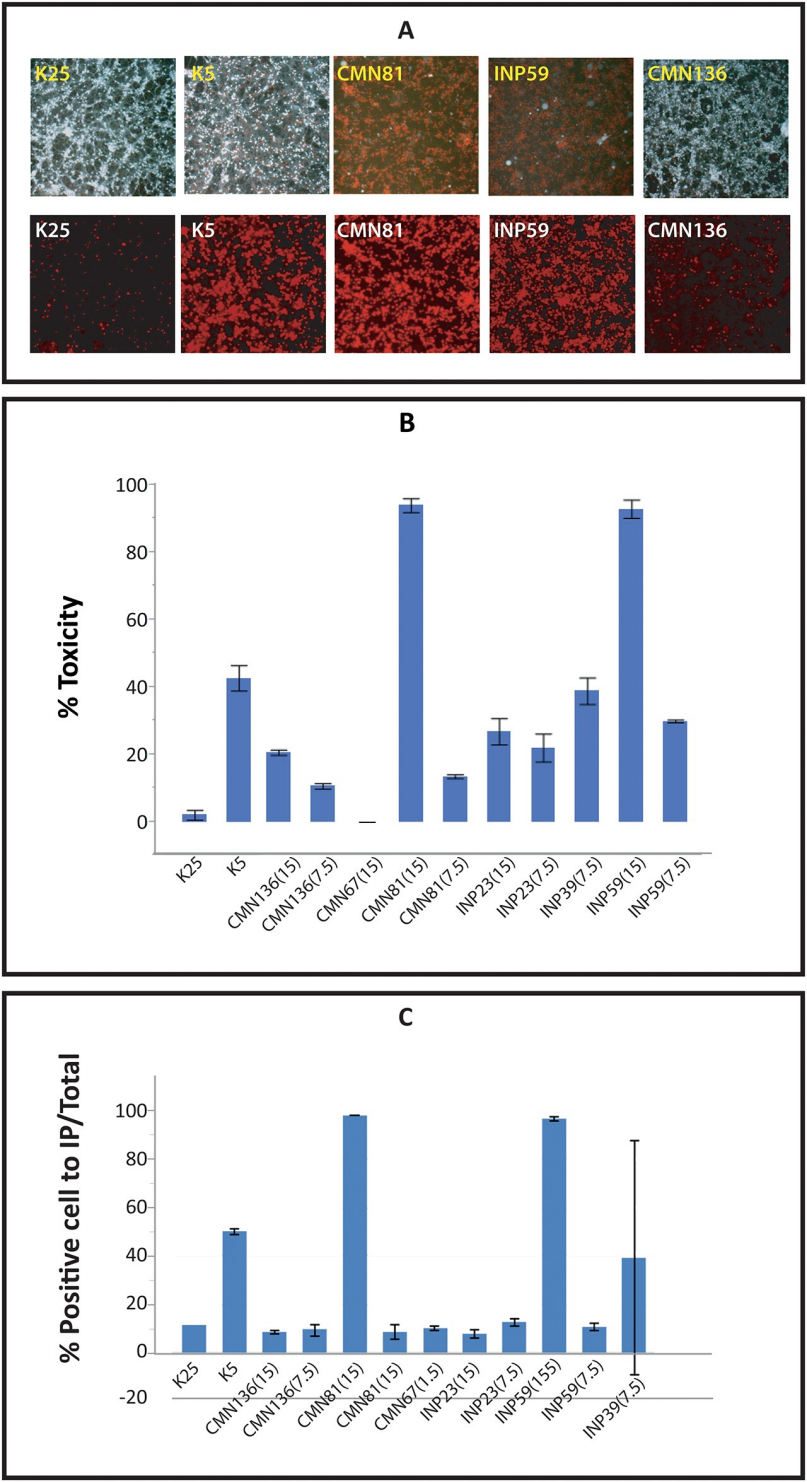
A. Neurotoxicity of RIAO from representative PTS of sera of patients and controls. Dose-dependent (7.5 = 0.029 mg/ml and 15 = 0.059 mg/ml) toxicity effects of RIAO on cerebellar granule cells (NGCs) as measured by the calcein-AM method. K25 is considered the optimal condition for NGC survival, while K5 induces more than 50% cellular death after 24 h. Codes of samples: CMN81:obesity; INP59:T1DM; CMN136:healthy child. B. Neurotoxicity of RIAO from representative PTS of patients and controls. Dose-dependent (7.5 = 0.029 mg/ml and 15 = 0.059 mg/ml) toxicity effects of RIAO on cerebellar granule cells (NGC) as measured by MTT. C. Neurotoxicity of RIAO from representative PTS of patients and controls. Dose-dependent (7.5 = 0.029 mg/ml and 15 = 0.059 mg/ml) toxicity effects of RIAO on cerebellar granule cells (NGC) as measured by calcein-AM methods. K25 is considered the optimal condition for NGC survival, while K5 induces more than 50% of cellular death after 24 h. Codes of samples: CMN136:healthy child; CMN81:obesity; CMN67:; INP23:T1DM; INP59: T1DM; INP39:T1DM.

Soluble amyloid oligomers of low molecular weight with a high surface hydrophobicity are the most cytotoxic because permeate the membrane and produce non-selective pores. In order to evaluate the toxicity of RIAO in more stringent conditions from representative PTS of sera of pediatrics patients and controls, we chose granular cells from the mouse cerebellum (CGC) (Fig 4).

We used two different approaches for the quantification of cell viability [33]. The first one used the accumulation of calcein-AM and exclusion of propidium iodide (IP) to stain live and dead cells, respectively. Fig 4A shows representative images of the 24 h treatment with different patients’ RIAO compared with NGC controls kept in 25 mM KCl. After this time, they were incubated with IP and calcein-AM for 15 min and were photographed with an epifluorescence microscope. The positive IP cells (dead) were counted in red because the IP binds to DNA and RNA and displays a red fluorescence signal. The viable cells were counted in green since they have enzymes that convert calcein-AM to calcein that display green fluorescence (Fig 4A–4C).

In addition, we assessed cell viability by the MTT (3-(4,5-dimethylthiazol-2-yl)-2,5-diphenyltetrazolium bromide) reduction technique. We considered the obtained result from the viable cells to be 100%, to which the results from different treatments were normalized. We found that, except for sample CMN67-T2DM, cellular death was induced by the RIAO to different degrees. In Fig 4B, we can see that CMN81-obesity and INP59-T1DM (at 15 concentration, for 24 h) had very strong toxic reactions. In the MTT evaluation, K25 is considered the optimal condition for NGC survival, while K5 induces more than 50% cellular death after 24 h [33].

In Fig 4A and 4C, we can see the effect of very high toxicity in CMN81-obesity and INP59-T1DM, while CMN136-healthy children showed no toxic effects (Fig 4).

### Overcome the biomarker dilemma in childhood obesity and DM

**Fig 5:**
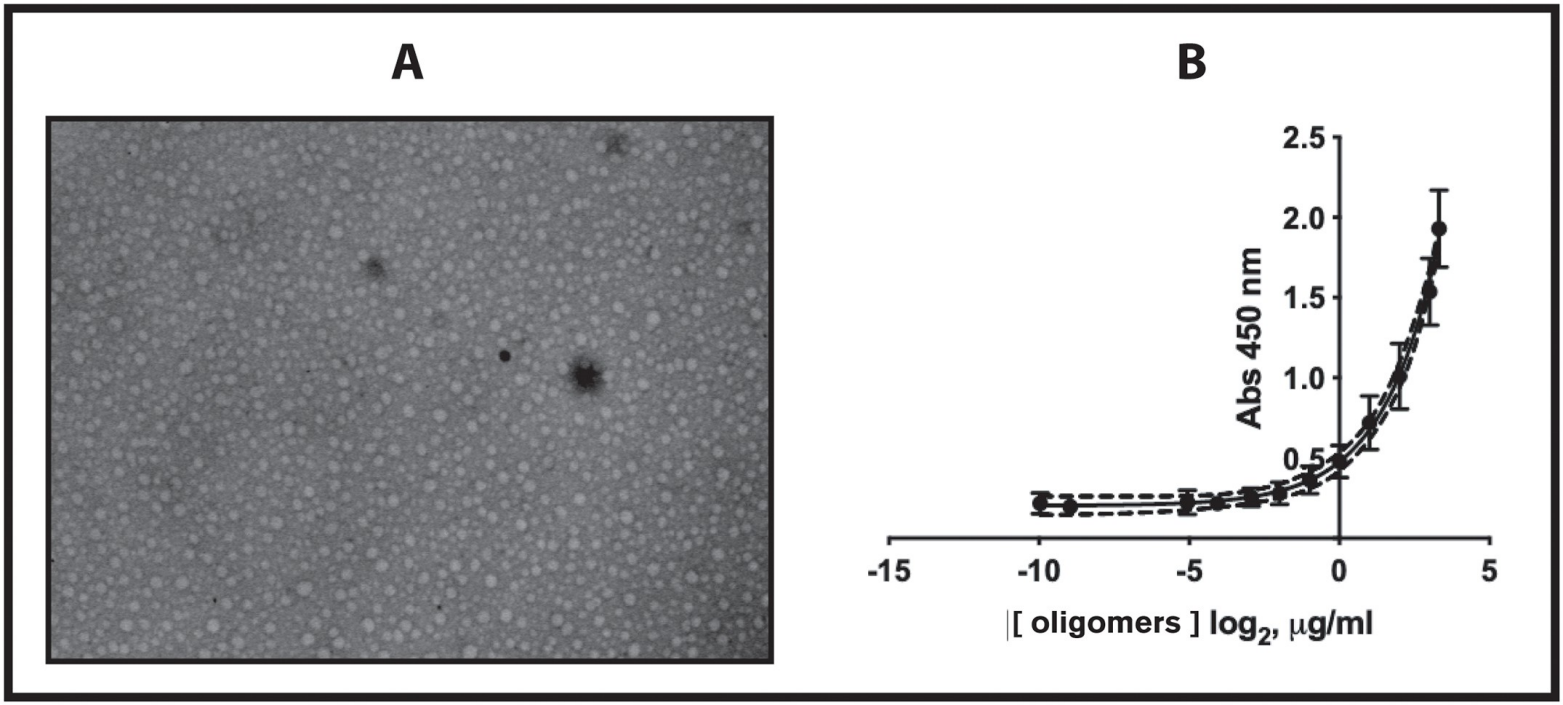
Standard curves of the new anti-hIAPP cytotoxic oligomer antibody’s reactivity to synthetic hIAPP cytotoxic oligomers. A). Representative negative stain TEM images of synthetic, spherical and cytotoxic oligomers of hIAPP used in the standard curves. B). The standard curves (n = 6) of polyclonal antibody reactivity to synthetic hIAPP cytotoxic oligomers were used to interpolate the hIAPP homo- and hetero-oligomer concentrations in the PTS of sera of children with obesity, type 1 diabetes and type 2 diabetes vs healthy children. R squared = 0.9524.

We overcame the biomarker dilemma by concentrating on oligomers with low molecular weight and cytotoxicity [20,38]. In order to overcome the limitations of ELISAs, we used a potential universal pretreatment that precipitated the oligomers (RIAO) as previously described [20,31]; it is cost-effective and easy to implement in diagnostic laboratories in hospitals. We performed ELISAs to confirm RIAO in the PTS from pediatric patients using the novel in-house anti-hIAPP cytotoxic oligomer antibody as described above (Fig 1C). The standard curve was performed using a synthetic hIAPP_1-37_ oligomer standards, prepared as described by Kayed to achieve the desired concentration of antigen [29]. The antigen was characterized by TEM (Fig 5A) and confirm its stability in 0.1% NH_4_OH (final pH 9.5–10.5) and stored a 4°C for months based on TEM and immunoassay experiments as described by our group and several groups for many amyloid oligomers [20,29,32].

The validation of standard curve was determined by the accuracy and reproducibility of the standard curve (running in triplicate) with 12 independent assays during the experimentation (on different plates, on different days, with different performers) under one single transformation measurement (approximately 1 year). The examples shown in Fig 5B are some of the curves made, and we found consistency between plates, as shown by the inter- and intra-assay variability.

We detected and quantified for the first time the presence of RIAO in 146 PTS patients from the different groups: 60 patients with T1DM (group A), 39 patients with T2DM (group B), 45 patients with obesity (group C) and 16 healthy children (control group D). RIAO in the PTS from sera are very stables in 0.1% NH_4_OH (final pH 9.5–10.5) and stored a 4°C for months; as well they are very stables in 50 mM sodium bicarbonate buffer (pH 9.0) when are freshly prepared, based on TEM, Circular dichroism and immunoassay experiments [20].

**Fig 6:**
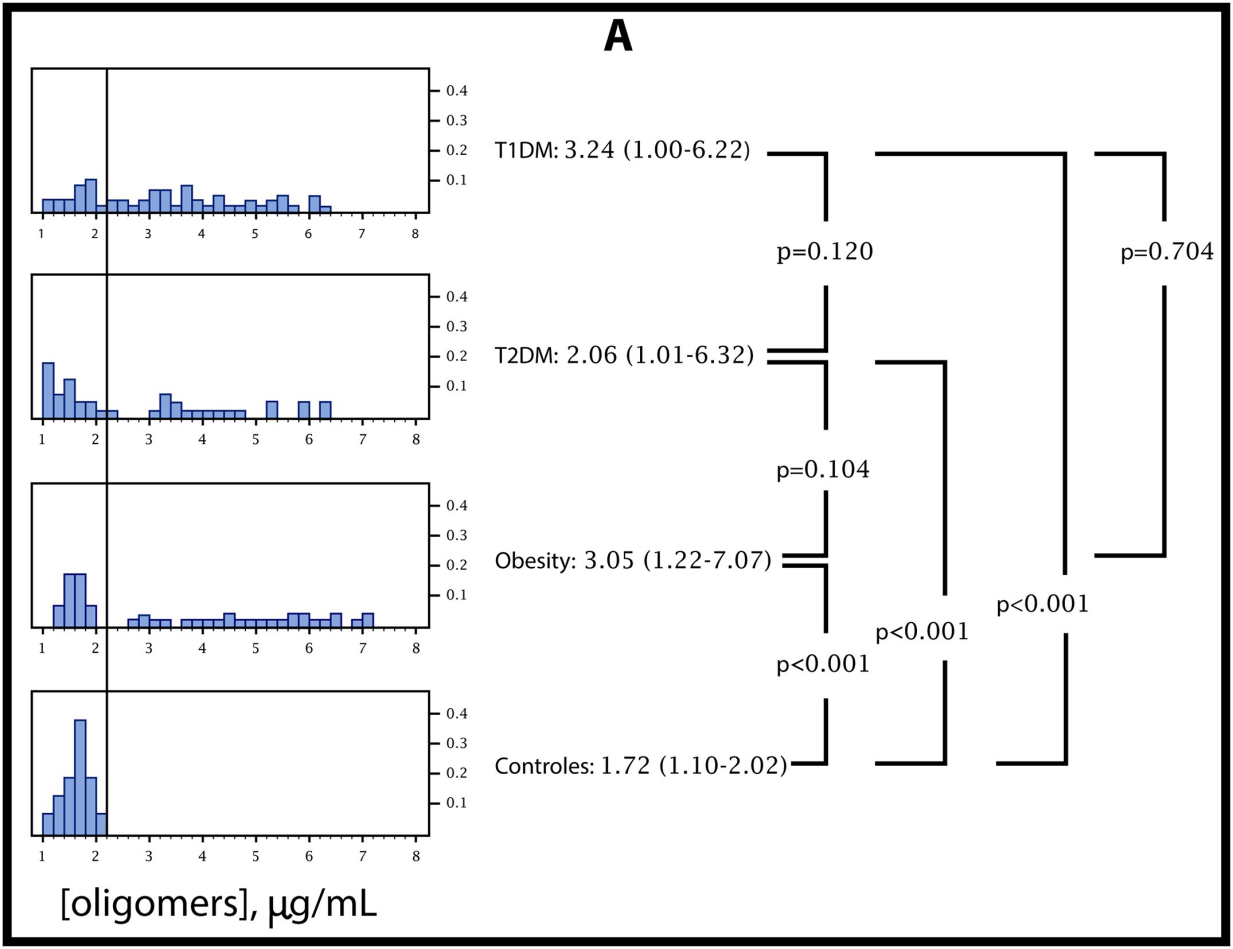
RIAO distribution of the four groups for visual comparison and the results of statistical analysis among groups by Welch’s test. Pairwise tests were done in a protected manner (one-way ANOVA with Welch’s correction: p<0.001).

We compared the levels of the cytotoxic oligomer between the four groups. The null hypothesis (equal distribution between the three groups) was rejected (Kruskal-Wallis: p<0.001). The distribution of RIAO levels of the four groups is presented. Visually, the difference in the shape of distribution between the healthy group D and the other three groups is evident. The statistical analysis showed a significant difference between the control group (1.6±0.3 μg/ml) and the other groups (in all the pairs p<0.001). No differences were found between the RIAO from patients with T1DM1 (3.4±1.5 μg/ml) and patients with T2DM (2.8±1.7 μg/ml) or obesity (3.6±2.0 μg/ml) p = 0.13 (Fig 6).

### RIAO are biomarkers of glucotoxicity in T1DM

We found a tendency to have higher RIAO levels when the evolution of T1DM was longer [β 0.0114 (0.0043) p = 0.011), with higher glucose levels [β (SE) 0.0071 (0.0020) p < 0.001) and higher levels of triglycerides [β (SE) 0.0057 (0.0016) p < 0.001)]. It was a striking result that in patients with more than 5 years of evolution and high glucose levels, the RIAO level increased [R^2^ = 0.17 (p = 0.002)].

We explored the interaction between the levels of glucose and HbA1c. We found that long-term evolution with poor glycemic control (HbA1C > 10%) was associated with fewer oligomers (p = 0.06). More than 7 years of evolution with poor glycemic control or hypertriglyceridemia correlated with a negative effect on the value of oligomers [R^2^ = 0.25 (p< 0.001) or R^2^ = 0.21 (p = 0.002)], respectively.

**Fig 7:**
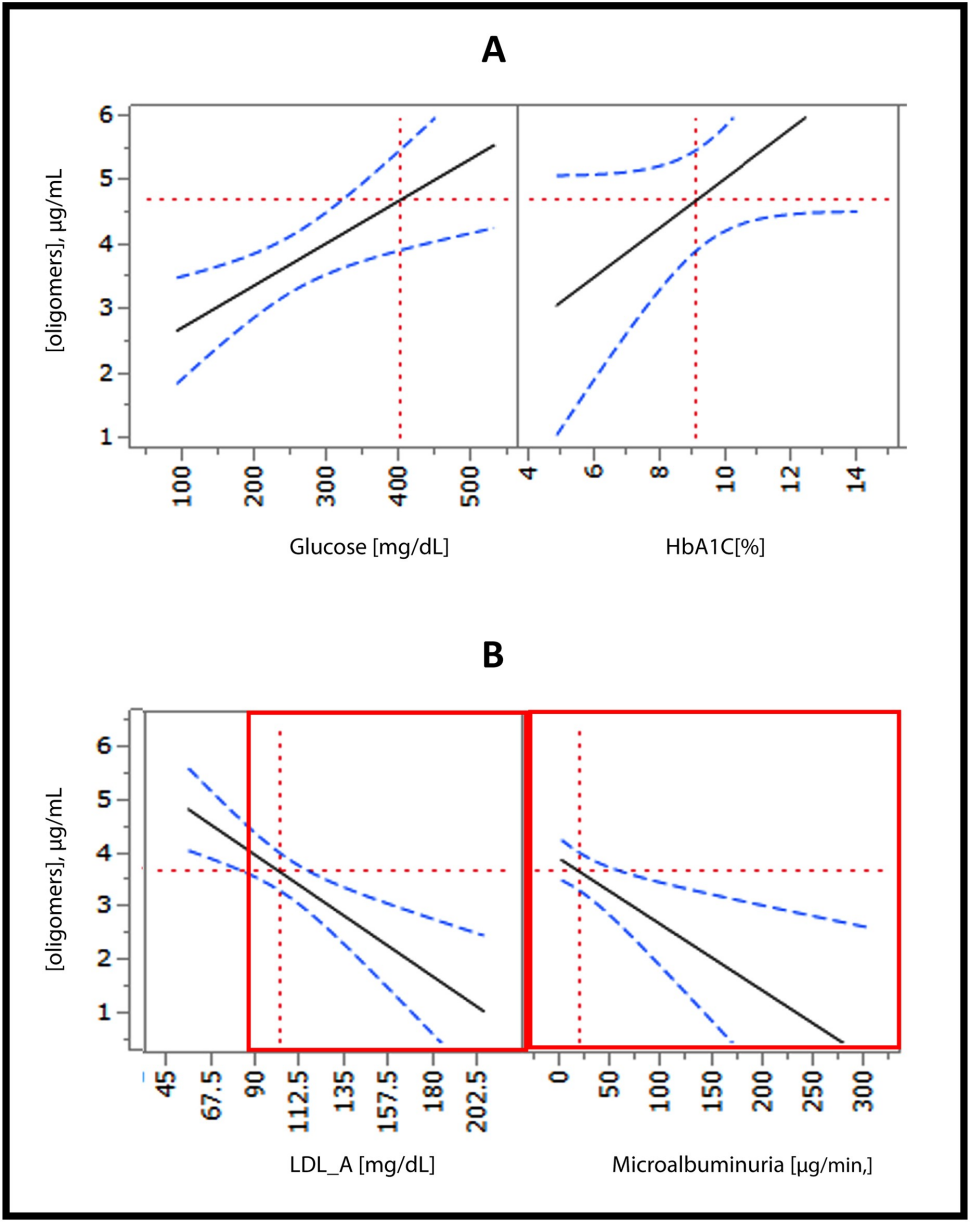
Linear regression analysis of the RIAO level and the values of glucotoxicity and its complications. A. The relationship between RIAO level and glucose or HbA1c: when glucose = 400 mg/dl or HbA1c = 9.072% then RIAO level = 4.72 μg/ml; [R^2^ = 0.70, p = 0.001]. B. The relationship between RIAO level and LDL or microalbuminuria; when LDL >100 mg/dl; or microalbuminuria >20 μg/min then RIAO level = 3.74 μg/ml; [R^2^ = 0.49, p = 0.001].

A linear regression model was used to measure the association of glucose level with RIAO level, conditioned by insulin dose. In patients with low doses (<0.6 UI kg/d), an association between glucose and oligomer levels was not observed. In patients requiring a high dose of insulin (> 0.7 UI kg/d), we found an association between higher glucose level and higher RIAO concentration [β (SE) 0183 (0.0049) p < 0.001)]. In patients with glucose <200 mg dL, a higher insulin dose was correlated with a lower RIAO concentration [R2 = 0.17, (p = 0.005)] (Fig 7).

### RIAO are modulated by cholesterol and lipotoxicity

The concentration of RIAO was diminished in T1DM patients with cholesterol > 170 mg/dl [β (SE) -0.0143 (0.0050) p = 0.06)] and when LDL >100 mg/dl (Fig 7B). These results are consistent with the findings of Singh and coworkers [39], who demonstrated a sevenfold decrease in amylin seeding on planar anionic membranes that contained cholesterol compared to those that lacked cholesterol. This finding is conspicuous since it reveals the intrinsic ability of cholesterol to regulate hIAPP oligomerization and deposition on membranes and protect cellular function. The role of cholesterol in modulating aggregation has been demonstrated in several amyloid proteins.

In the logistic regression analysis, the interaction between RIAO level in T1DM patients with zBMI > 2 SD and uric acid >5.4 mg/dL showed a low RIAO value [R^2^ = 0.26 (p = 0.002)].

In terms of the evolution of disease associated with TG level, RIAO had greater concentrations in patients with T1DM for less than 4 years and higher TG values, but in patients with T1DM for more than 7 years and higher TG levels, RIAO concentrations were low [β (SE) -0.0001 (4.9x10^-5^) p = 0.005].

In children with T2DM, dyslipidemia (high CT, LDL, and non-HDL, and low HDL) was associated with low RIAO levels, especially high non-HDL and hypertriglyceridemia (Table 2).

**Table 2:** General lineal model: Variables associated with the level of RIAO in patients with TIDM.

	*β* (SE)	*t*-ratio	P-value
Intercept	4.7708 (0.7720)	6.18	<0.001
Months evolution	0.0076 (0.0043)	1.76	0.083
Triglycerides	0.0049 (0.0017)	2.93	0.005
Colesterol	-0.0133 (0.0051)	-2.59	0.012
Months evolution*Triglycerides	-0.0001 (5.2x10^-5^)	-2.27	0.027

*β*: regression coefficient, SE: standard error. R^2^ adjusted = 0.21, Total model: P<0.001

### Obesity as a protein conformational disease in childhood: Novel classification

We also carried out an integral study of the clinical data and used an immunoassay that is not only simple—when coupled with a well-taken patient’s medical history—but also cost-effective and able to quantify the presence of RIAO.

In the population of children with obesity, the median RIAO score was significantly higher than in healthy children [3.1 (1.2–7.1) vs 1.7 (1.1–2.0), p < 0.001]. We compared the values of RIAO with the currently accepted indicators of β-cell function and glucotoxicity [40–43] by simple linear regression: glucose had a tendency to be associated with higher RIAO (p = 0.119); HbA1C, C-peptide and HOMA had a positive correlation, with p = 0.027, p = 0.017, and p = 0.012, respectively.

We observed that the number of RIAO increased as the number of complications increased, and significantly so when a child with obesity presented any of the following comorbidities: glucose > 85 mg/dL but < 100 mg/dL, insulin resistance (HOMA and C-peptide), dyslipidemia (hypertriglyceridemia, hypoalphalipoproteinemia), hyperuricemia, alanine amino transferase (ALT) elevation and/or diastolic hypertension, (p = 0.003). Our analysis supports this hypothesis if a child presented four or more complications; the median of RIAO was greater in these children than in children with two or fewer (median: 4.52 [IQR: 2.23], 1.62 [IQR: 1.49], Welch test: p = 0.003). If a child had three or more comorbidities, the median OC was greater than in children with two or fewer comorbidities (medians: 5.02 [IQR: 2.3], 1.71 [IQR: 3.1], Welch test: p <0.001).

**Fig 8:**
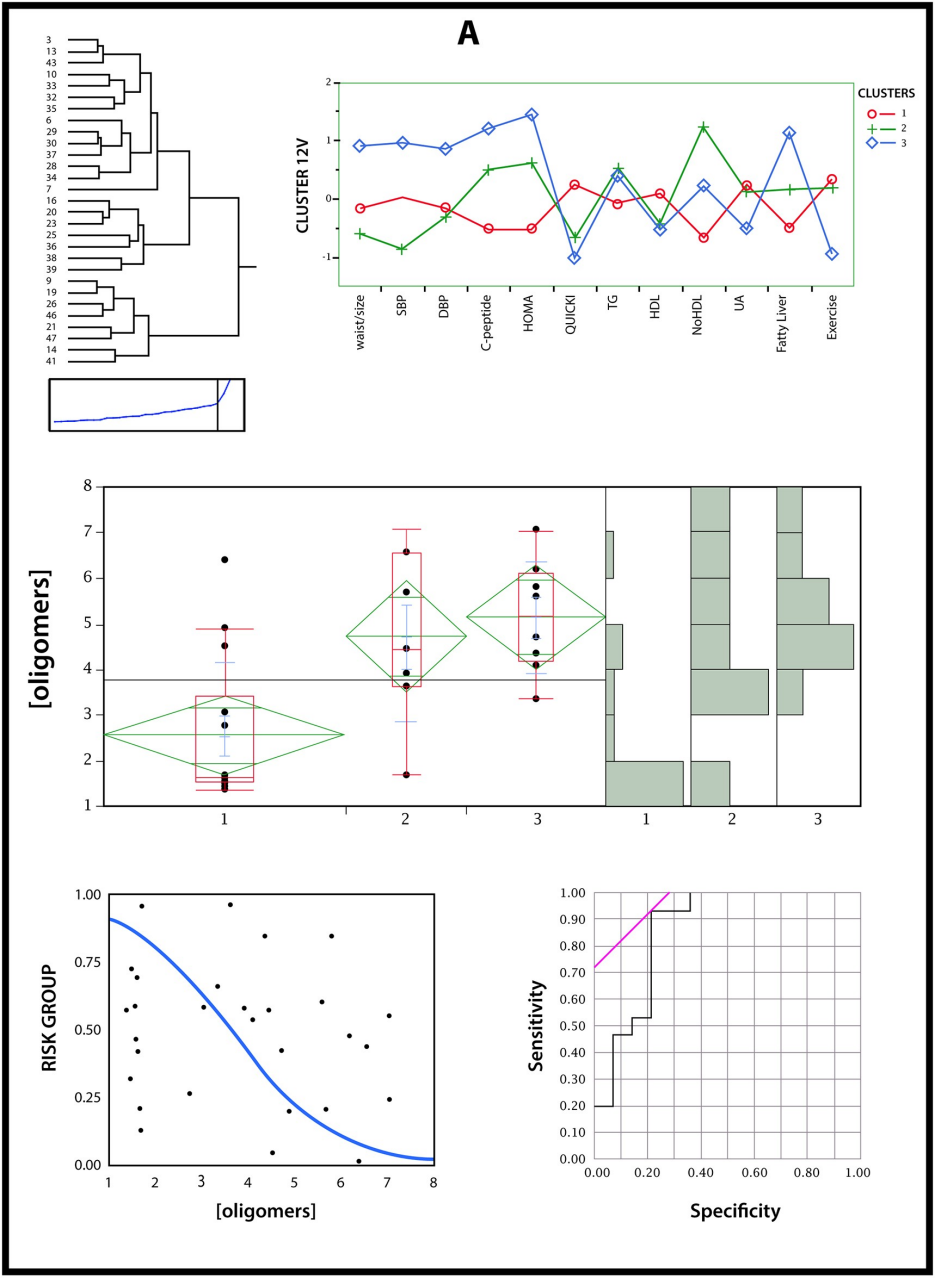
Data-driven cluster analysis to identify cases with a high risk of beta cell damage. A cluster analysis of several variables that were related to beta cell state was performed for patients with obesity (12 variables); by means of a cluster analysis, 3 subgroups were obtained whose profiles can be typified as “healthy” obesity, gynecoid obesity, and android obesity. The ROC curve for patients with obesity displays a sensitivity of 0.93 and specificity of 0.79. Lower RIAO values (< 3.35 μg/ml) suggest integrity of beta cell functions (p < 0.001). The cut point expresses the increase in risk [OR de 51.32 [4.67–564.16] p = 0.001] for children with a positive result in comparison to those with a negative result (<3.35 μg/ml).

We examined whether the RIAO level was tagged with clinical-biochemical profiles (waist/size ratio, SBP and DBP centile, C-peptide in fasting, HOMA, QUICKI, TG, C-HDL, C-NoHDL, UA, ALT, and exercise) using data-driven cluster analysis; 3 clusters were obtained whose profiles could be typified as “healthy” obesity (cluster 1), gynecoid obesity (cluster 2), and android obesity (cluster 3) (Fig 8). A data-driven cluster analysis of 12 variables (waist/size ratio, SBP and DBP centile, C-peptide in fasting, HOMA, QUICKI, TG, C-HDL, C-NoHDL, UA, ALT, and exercise) measured at the time of the study in children (n = 47) identified three clusters of patients with different profiles of high risk of β cell damage. One cluster was associated with a low RIAO level, and two clusters had high RIAO values (medium (minimum, maximum); they were 4.45 μg/ml (1.71, 7.07), 5.17 μg/ml (3.35, 7.05) and 1.65 μg/ml (1.39, 6.40), respectively (Welch’s test: p = 0.003, comparison by pairs protected by the global test was significant between gynecoid obesity or android obesity and "healthy" obesity at the level of p <0.05) (Fig 8).

We next explored the connection between RIAO values for the low-risk cluster vs the high-risk clusters by means of logistic regression. The RIAO level was used as an independent variable, and the high-risk vs low-risk clusters were used as the dependent variable. This model showed a clear association between the two variables (Wald´s chi-squared = 12.93, p < 0.001).

Based on this model, a ROC curve was obtained to determine the cut point of the RIAO level for predicting high-risk cases. We found that a RIAO level of 3.35 μg/ml was the cut point that optimized the parameter estimations in the diagnostic test: AUC = 0.86 [IC95%: 0.76–0.96]; exactitude (overall accuracy) = 0.86 [IC95%: 0.74–0.99]; sensitivity = 0.93 [IC95%: 0.81–1.00]; specificity = 0.79 [IC95%: 0.57–1.00]; positive likelihood ratio = 4.37 [IC95%: 1.58–11.98]; negative likelihood ratio = 0.08 [IC95%: 0.01–0.57] (Fig 8).

Two types of ORs for high risk of β-cell damage were calculated: one was due to a one-unit in RIAO, and the other for presenting an RIAO level higher than the identified cut point of 3.35 μg/ml.

We explored lipotoxicity in the obesity group in patients with a high risk of β-cell damage with RIAO values > 3.35 μg/ml: higher triglycerides [155 (IQ 104) vs 107 (IQ 83), p = 0.041]; lower HDL cholesterol- [37.5 (IQ 9) vs 45.2 (IQ 14), p <0.001] and higher ALT [31.6 (IQ 14) vs 19.6 (IQ 8), p = 0.002] (Fig 8).

### Further insights into pediatric type 2 diabetes as a protein conformational disease: Novel subgroups

**Fig 9:**
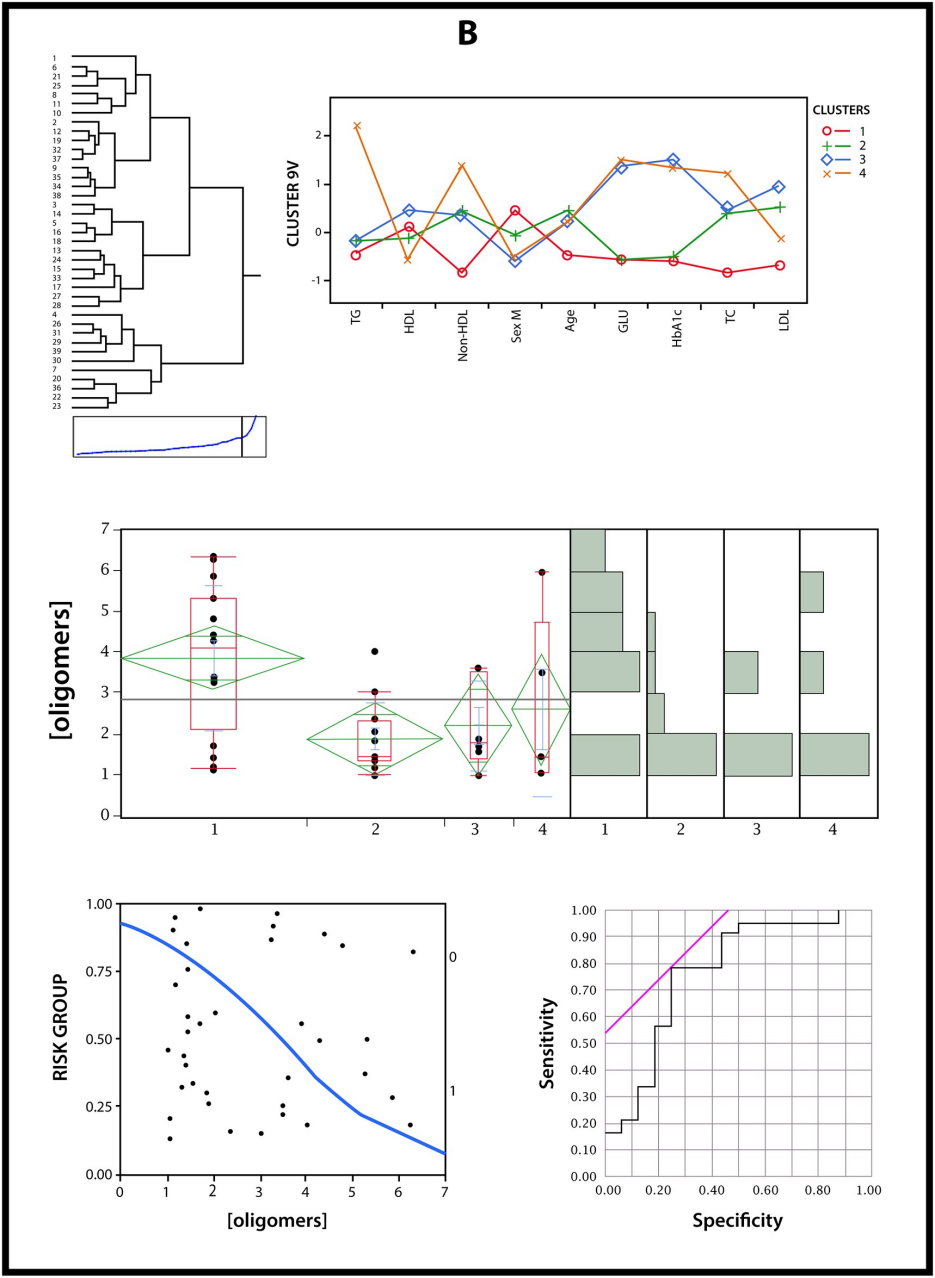
Data-driven cluster analysis to identify cases with a high risk of beta cell damage. A cluster analysis of 9 variables for patients with T2DM that were related to beta cell state. The figure show the clusters and their relationships with the RIAO level and finally the cluster-specific receiver operating characteristic (ROC) curve; by means of a data-driven cluster analysis, 4 subgroups were identified: cluster 1 labeled T2DM with good metabolic control; cluster 2 labeled T2DM with good glycemic control but with dyslipidemia; cluster 3 labeled T2DM with bad metabolic control; and cluster 4 labeled T2DM with bad metabolic control and substantially higher TG and non-HDL levels. The ROC curve for patients with T2DM had an elevated AUC value (0.77 [95% CI: 0.65, 0.89]), which generated the RIAO cut point of 3.03 μg/ml that optimizes the relation of sensitivity and specificity.

In the T2DM group (group B), using the data-driven cluster analysis, we identified 4 clusters: cluster 1 labeled T2DM with good metabolic control; cluster 2 labeled T2DM with good glycemic control but with dyslipidemia; cluster 3 labeled T2DM with bad metabolic control; and cluster 4 labeled T2DM with bad metabolic control and substantially higher TG and non-HDL levels (Fig 9). Cluster 1 had the highest RIAO levels in relation to clusters 2 and 3: 4.09 μg/ml (1.13, 6.32) vs 1.44 μg/ml (1.05, 5.95) (p<0.001) vs 1.79 μg/ml (1.01, 3.62) (p = 0.019). Between clusters 1 and 4, no statistically significant difference was detected (p = 0.277), although the RIAO distribution was lower (1.05, 5.95) (Fig 9).

The patients from clusters 3 and 4 received treatment with exogenous insulin (88% and 80%, respectively), while in cluster 2, only 17% did, and cluster 1, just 1.31% did. Good glycemic control was associated with higher RIAO levels.

Then we performed an analysis with a logistic regression model, with the group of children with greater damage in β-cells as an independent variable, and it yielded high statistical significance (Ward test: chi-squared = 10.73, p = 0.001). A corresponding ROC curve was calculated, which had an elevated AUC value (0.77 [95% CI: 0.65, 0.89]) that generated a RIAO cut point of 3.03 μg/ml that optimized the relationship between sensitivity and specificity. The accuracy parameters were as follows: sensitivity = 0.78 [95% CI: 0.61, 0.95]; specificity = 0.75 [95% CI: 0.54, 0.96]; positive likelihood ratio = 3.13 [95% CI: 1.30, 7.51]; and negative likelihood ratio = 0.29 [95% CI: 0.13, 0.66]. With the same model, the odds ratio that corresponded to each one-unit change in RIAO was 2.04 μg/ml [IC95%: 1.25–3.33], and the model to evaluate the association of the dichotomized RIAO by the cut point obtained with the risk group generated the odds ratio of 10.80 μg/ml [IC95%: 2.40–48.60] (p = 0.002).

## Discussion

The prevalence of childhood obesity and diabetes is increasing in the world [44]. The large variations of prediabetes and DM in several countries is a challenge for the healthcare system revealing that obesity and DM are catastrophic and harrowing diseases [3,27,44]. By using real patient´s samples we began to unravelling the role of RIAO and we are advancing toward the novel diagnostic tools, prevention strategies and novel therapeutic targets [9,20,30]. The biomarkers of β-cell failure were until now an elusive task, the most being used and/or studied in pediatric population are those that concern local or systemic inflammation processes, oxidative stress, as well as those related to endothelial dysfunction processes; many of them are low intensity marker inasmuch as its elevation has a wide differential diagnosis [45–49].

Here, we present our findings from a cross-functional translational and independent studies showing that RIAO are elevated in serum of childhood obesity and diabetes; we also carried out an integral study of the clinical data and used an immunoassay that is not only simple—when coupled with a well-taken patient’s medical history—but also cost-effective and able to quantify the presence of RIAO and demonstrated that its work as biomarkers of early β-cell failure.

**Fig 10:**
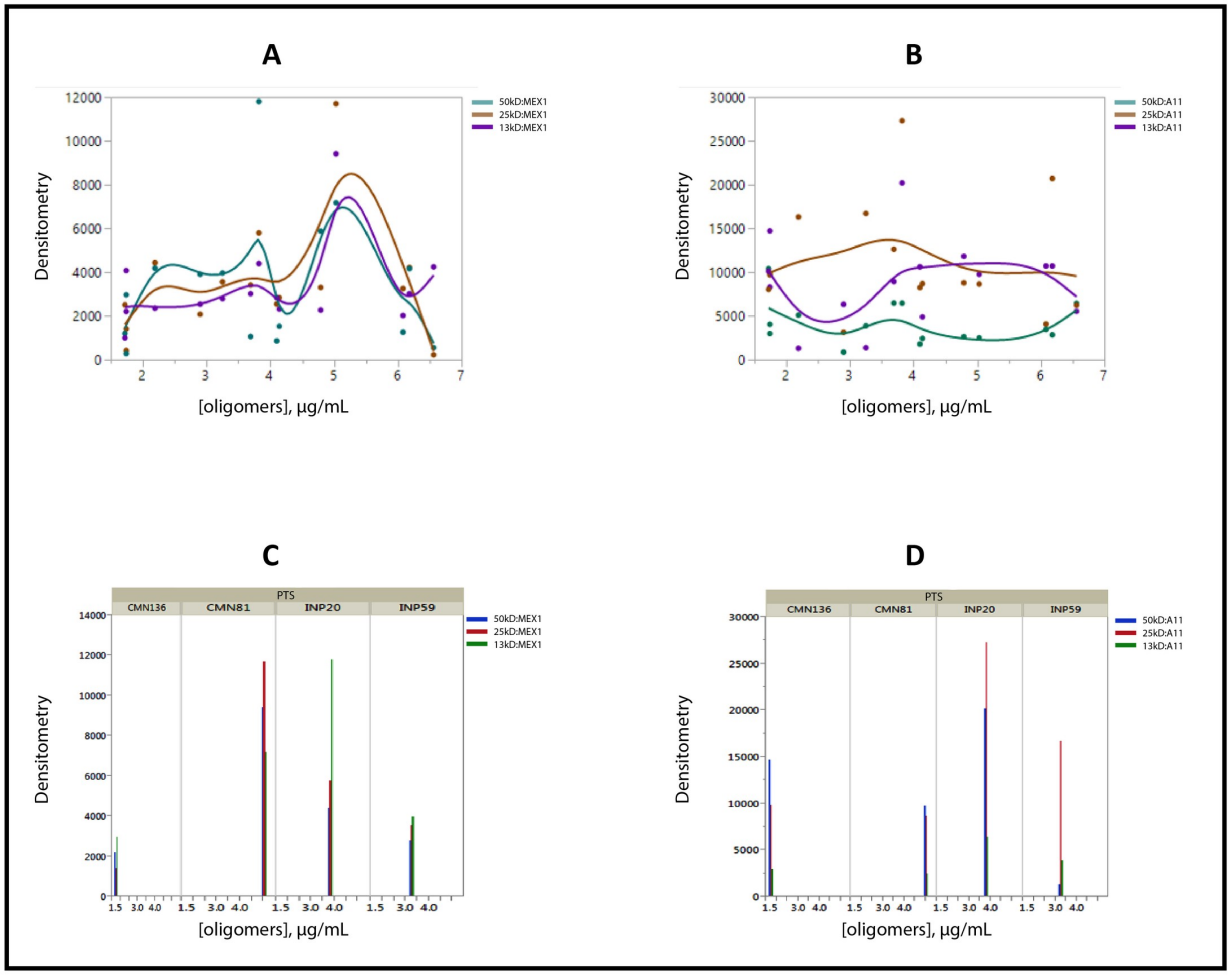
Bivariate fit of Immunoblotting (densitometry, AU) by RIAO levels in PTS. A. MEX1 antibody: smoothing spline fit, lambda = 0.01; B. A11 antibody: smoothing spline fit, lambda = 0.01. C and D. Representative patient´s samples (CMN81:obesity; INP20:T1DM; INP59:T1DM and control sample (CMN:136).

To determine a possible correlation between immunoblotting with MEX1 or A11 antibodies (Densitometry values, A.U) and RIAO level measured by indirect ELISA a bivariate analysis was performed (Fig 10). A striking result is the augmentation of all oligomeric species ranging from 13 kDa and above when the RIAO level is higher in PTS. We want to point out in Fig 10A and 10B, the present of protein band of 25 kDa which corresponds to homo-hexamer and it is the highest band when the level of RIAO are elevated. This result unravelling the role of the hIAPP homo-hexamers in childhood obesity and diabetes and together with the results obtained by several studies as a relevant step of aggregation competent state in which: i) oligomerization starts from hexamers [50,51]; ii) the hexamer are biomarkers and can co-aggregated with several proteins [9], iii) the identification-structural characterization of the hexamer oligomers required for the protein DN6 oligomerization [52] and the demonstration of hIAPP are homo and hetero-oligomers in real patient´s samples, constituted a strong evidences in the discovering of new diagnostic and therapeutic targets in obesity and diabetes as PCD [30,53–57] (Fig 10A and 10B). One way of explaining the densitometric distribution differences in the reactivity of the PTS with MEX1 and A11 antibodies is RIAO level of each patient´s PTS (Fig 10C and 10D).

We opens novel epistemic horizons that will contribute to changing our understanding about the PCD in children and adolescents [20,39,58]. This has been done by accelerating the unpacking of the aggregation-oligomerization-fibrillization process in real patient´s samples and uncovering novel physico-chemical features of RIAO and their ipso facto application to the clinical field [20]. New paradigms have emerged in the process: first, obesity, T1DM, and T2DM are PCD [18,20,28,42,59,60]. Defying the beliefs of age-dependent risk [61], our results demonstrated that children form RIAO and fibrils.

**Fig 11:**
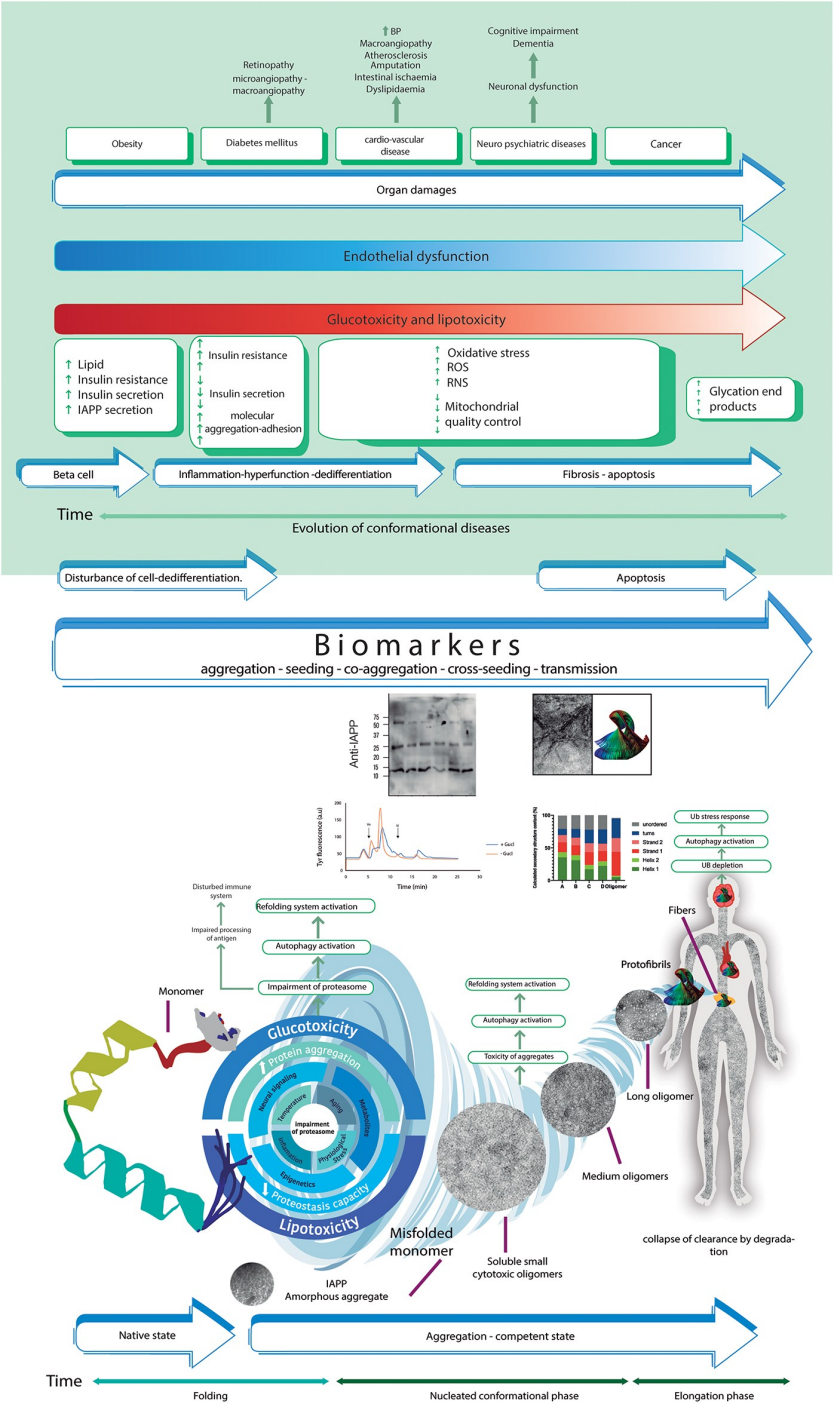
The proposed novel physio-pathological pathway of the hIAPP aggregation-oligomerization-fibrillization process in childhood obesity and DM as a protein conformational disease. hIAPP undergoes conformational changes and enters an aggregation-competent state (misfolded), increasing protein aggregation and decreasing the capacity for proteostasis, leading to self- and hetero-oligomerization and, finally, conversion into hIAPP fibers. The cytotoxic oligomers cause apoptosis in several cells and the appearance of cross-seeding amyloid structures. At the cellular level, the aggregation-oligomerization-fibrillization process reflects a disturbance of the cell, dedifferentiation and finally apoptosis. The metabolic changes that accompany this process are multidimensional, resulting in glucotoxicity and lipotoxicity that, in conjugation with RIAO, are responsible for organ damage and the appearance of the comorbidities obesity, DM, cardiovascular disease, neurological disease, and many cancers.

We first undertook isolation-stabilization, initial ultrastructural morphological-immunoreactivity, and biophysical studies of RIAO [20]. Second, RIAO levels in PTS from children allowed us to propose a novel classification of these PCD by clustering patients in a way that identified children at high risk of β cell failure and other comorbidities (Figs 1–10) and their physiopathological pathways. Fig 11 summarizes the relevant steps from the basic research knowledge to the clinical outcomes by combining the results from our group and the scientific literature [3,18,60,62–68].

We showed that the aggregation-oligomerization-fibrillization process is real and occurred in the patient´s samples; monomeric hIAPP generated soluble aggregates that had conformational changes (as shown by circular dichroism studies) and acquired a cross-β-structure to become compact-stable (small, medium and large oligomers), as observed by TEM [20]. Real hIAPP amyloid oligomers, which, are able to form fibers and fiber networks (TEM and ThT experiments). RIAO and fibrils react with anti-hIAPP, anti-oligomer (A11) and anti-hIAPP cytotoxic oligomer antibodies (MEX1), (TEM immunolocalization experiments and immunoassay detection); RIAO are human homo- and hetero-oligomers, as demonstrated by HPLC and mass spectrometry in our previous studies [20]. They are cytotoxic to cerebellar neurons (MTT and calpain experiments) (Fig 4).

RIAO have to trigger all autophagy and refolding mechanisms to maintain the proteostasis balance (as suggested by the presence of immunoglobulins and haptoglobin in the RIAO) [20].

In this paper we demonstrated that human RIAO circulate throughout the body (Figs 1–11), causing cellular death by two mechanisms: interacting with membranes to produce disruption, as demonstrated *in vivo* and *in vitro* in several papers [69,70], and replacing islet cells with amyloid deposits, which appear in the pancreas in 90% of patients autopsied with T2DM [21]. Most of the molecules that are involved in the aggregation-oligomerization-fibrillization process and its control are potential biomarkers (Figs 7–11), [9,71,72]. We were able using a sound and reliable Elisa assay to identify the RIAO cut point (≥ 3.35 μg/ml) on the AUC curve as a biomarker of greater β cell damage in children with obesity and high cardiometabolic risk (severe comorbidities: dyslipidemia, fasting hyperglycemia, fatty liver disease elevation) or functional alteration of β cells (resistance to insulin HOMA and fasting C peptide), with a sensitivity of 0.93 and specificity of 0.79. Lower RIAO values (< 3.35 μg/ml) suggest the integrity of β cell functions (p < 0.001). The cut point expresses the increase in risk [OR de 51.32, p = 0.001] for children with a positive result in comparison to those with a negative result (<3.35 μg/ml) (Figs 8–10).

The cooperative effect of co-aggregation and cross-seeding observed by several authors and us reveals the complexity of the process [73,74]. At the cellular level, all the factors described above reflect disturbance of the cell, dedifferentiation and finally apoptosis (Figs 4 and 11). The metabolic changes that accompany this process are multidimensional, resulting in glucotoxicity and lipotoxicity (Figs 7–9 and Table 2) that, in conjugation with human homo and hetero-RIAO, are responsible for organ damage and the apparition of comorbidities (obesity, DM, cardiovascular diseases, neurological diseases, and many cancers) (Figs 7–11).

Our understanding of the amyloid biomarkers in relation to obesity and DM is in evolution. Furthermore, because of the hetero-oligomeric nature of hIAPP *in vivo*, all of the studies carried out in amyloid oligomers have used synthetic hIAPP. This is the first study done with naturally occurring or real hIAPP oligomers (RIAO) and a successful translation from basic science into applied science.

In clinical practice, the human homo and hetero-RIAO are useful for identifying cases with β cell damage and could substitute for many clinical and biochemical measurements (Figs 7–10). Because the diagnostic accuracy parameters presented wide confidence intervals, this method does not allow the RIAO cut point to be the recommended value for clinicians. Nevertheless, the results were encouraging for the next step of our translational process, which will be to perform a larger study focusing on children with obesity without DM. RIAO could have a fundamental role in differentiating between children with obesity with a high and a low risk of β-cell failure. This would change the initial diagnosis of the child and would result in a decrease in the treatment costs of the whole biochemical analysis by only obtaining the RIAO level instead of 22 other variables, and it would indicate whether obesity is associated with a metabolic syndrome or not [5,6]. An essential factor in the low RIAO levels for children with obesity is exercise (Fig 9). This enforces the importance of physical activity as a decisive element for reducing cardiometabolic risk [75].

Today, as biomedical and clinical practice scientific-community we have to inform and raise awareness in the scientific community and the health care institution on the nature-scope of RIAO and their potential role as a vector molecules in the prion-like transmission [76,77] of the obesity and diabetes in particular and the PCD in general [18,78,79].

This study tackles the challenge in translational medicine of finding more efficient strategies for understanding, diagnosing, preventing, controlling, testing the transmission mechanisms of, and treating childhood obesity and DM as PCD that can be applied in open populations [30,53,57,80,81].

## Supporting information

S1 File (DOCX)
