# Supplementary material for: Protein-conformational diseases in childhood: Naturally-occurring hIAPP amyloid-oligomers and early β-cell damage in obesity and diabetes
Source: PLoS One. 2020 Aug 24;15(8):e0237667. doi: 10.1371/journal.pone.0237667 (PMC7446879; doi:10.1371/journal.pone.0237667)

**Protein-conformational diseases in childhood: naturally-occurring hIAPP amyloid-oligomers and early β−cell damage in obesity and diabetes**

Nelly F. Altamirano-Bustamante^1¶^, Eulalia Garrido-Magaña^2¶^, Eugenia Morán^3^, Aurora Calderón ^3^, Karina Pasten-Hidalgo ^1,4^, Rosa Angélica Castillo-Rodríguez^1,4^, Gerardo Rojas^2^, Reyna Lara-Martínez^5^, Edgar Leyva-García^3^, Mateo Larralde-Laborde^3^, Guadalupe Domíguez^6^, Chiharu Murata^1^, Yolanda Margarita Vazquez^1^, Rafael Payro^3^, Manuel Barbosa^3^, Alejandro Valderrama^1^, Hortencia Montesinos^1^, Alejandra Domínguez-Camacho^1^, Víctor H. García-Olmos^1^, Regina Ferrer^3^, Patricia G. Medina-Bravo^7^, Fernanda Santoscoy^3^, Cristina Revilla-Monsalve^3^, Luis Felipe Jiménez-García^5^, Julio Morán ^6^, Jalil Villalobos Alva^3^, Mario Javier Villalobos^3^, Raúl Calzada-León^1^, Perla Altamirano^3^, Myriam M. Altamirano-Bustamante^3 ¶,*^

^1^Instituto Nacional de Pediatría, Mexico city, Mexico.

^2^UMAE Hospital de Pediatría, Centro Médico Nacional Siglo XXI, Instituto Mexicano del Seguro Social. Mexico city, Mexico.

^3^Unidad de Investigación en Enfermedades Metabólicas, Centro Médico Nacional Siglo XXI, Instituto Mexicano del Seguro Social, Mexico city, Mexico.

^4^Cátedras Conacyt.

^5^Facultad de Ciencias, UNAM, Mexico city, Mexico.

^6^Instituto de Fisiología Celular, UNAM, Mexico city, Mexico.

^7^Hospital Infantil Federico Gómez, Mexico city, Mexico.

^*^Corresponding author:

Email: [myriamab@unam.mx](mailto:myriamab@unam.mx). (MMAB)

^¶^These authors contributed equally to this article.

**SLOT BLOT anti-hIAPP cytotoxic oligomer prepared in house**


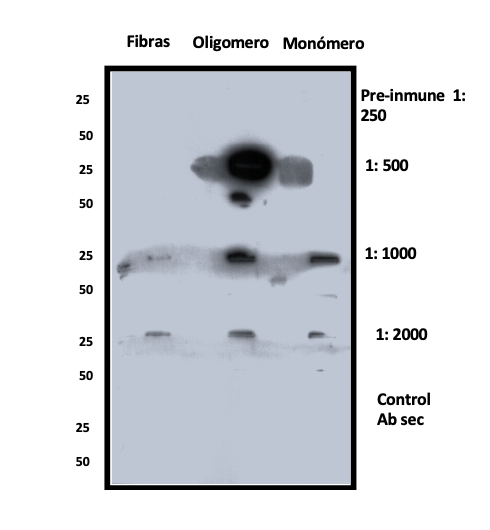


WB of patient´s PTS with anti-hIAPP cytotoxic oligomer


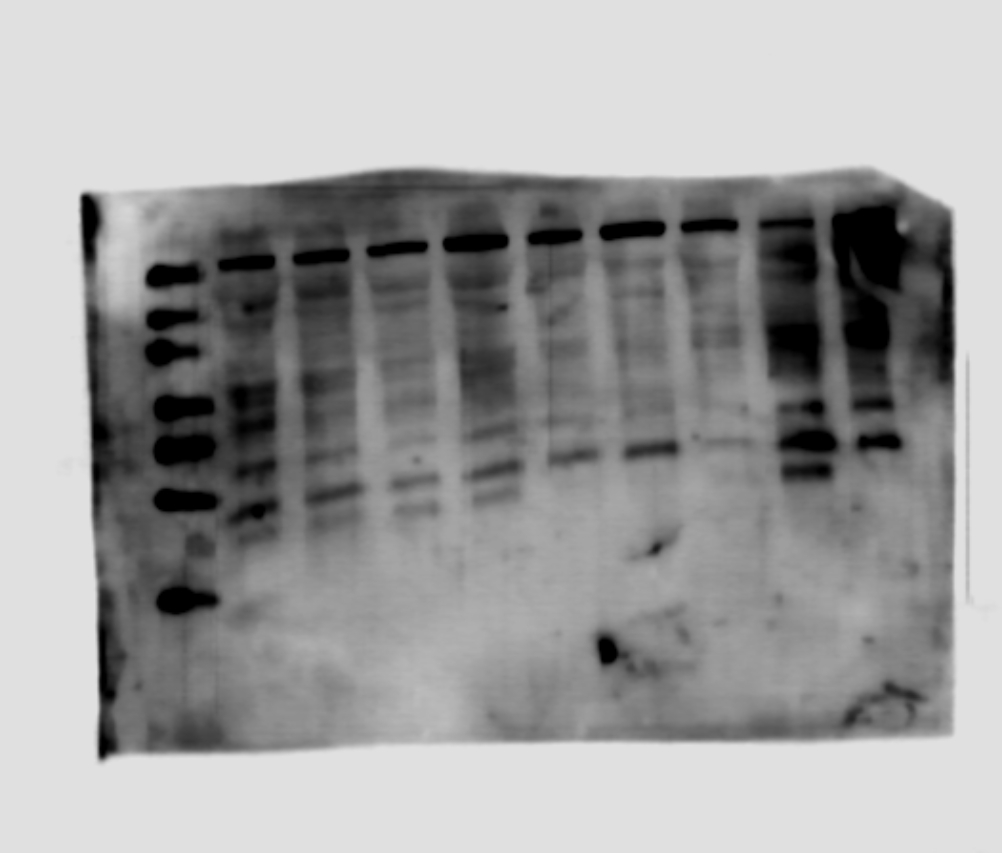


WB of patient´s PTS with A11


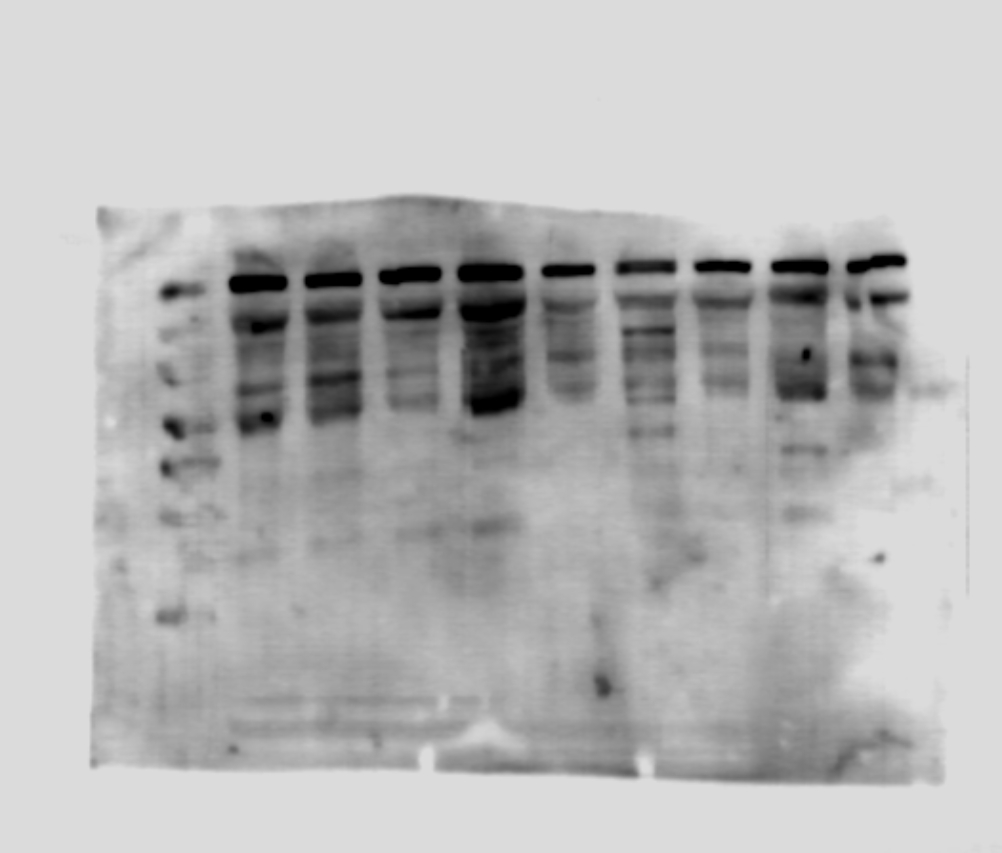

Supplement: S1 File — (DOCX) [file pone.0237667.s001.docx]
